# A genome-wide data assessment of the African lion (*Panthera leo*) population genetic structure and diversity in Tanzania

**DOI:** 10.1371/journal.pone.0205395

**Published:** 2018-11-07

**Authors:** Nathalie Smitz, Olivia Jouvenet, Fredrick Ambwene Ligate, William-George Crosmary, Dennis Ikanda, Philippe Chardonnet, Alessandro Fusari, Kenny Meganck, François Gillet, Mario Melletti, Johan R. Michaux

**Affiliations:** 1 Barcoding of Organisms and tissues of Policy Concern (BopCo)/Joint Experimental Molecular Unit (JEMU), Royal Museum for Central Africa, Tervuren, Belgium; 2 Conservation Genetics, Department of Life Sciences, University of Liège, Liège, Belgium; 3 Wildlife Division, Ministry of Natural Resources and Tourism, Dar es Salaam, Tanzania; 4 Fondation Internationale pour la Gestion de la Faune (IGF), Paris, France; 5 Tanzania Wildlife Research Institute, Arusha, Tanzania; 6 Barcoding of Organisms and tissues of Policy Concern (BopCo), Royal Museum for Central Africa, Tervuren, Belgium; 7 African Buffalo Initiative Group (AfBIG), IUCN/SSC/ASG, Rome, Italy; 8 Centre de Coopération Internationale en Recherche Agronomique pour le Développement (CIRAD), UPR AGIRS, Campus International de Baillarguet, Montpellier, France; National Cheng Kung University, TAIWAN

## Abstract

The African lion (*Panthera leo*), listed as a vulnerable species on the IUCN Red List of Threatened Species (Appendix II of CITES), is mainly impacted by indiscriminate killing and prey base depletion. Additionally, habitat loss by land degradation and conversion has led to the isolation of some subpopulations, potentially decreasing gene flow and increasing inbreeding depression risks. Genetic drift resulting from weakened connectivity between strongholds can affect the genetic health of the species. In the present study, we investigated the evolutionary history of the species at different spatiotemporal scales. Therefore, the mitochondrial cytochrome *b* gene (N = 128), 11 microsatellites (N = 103) and 9,103 SNPs (N = 66) were investigated in the present study, including a large sampling from Tanzania, which hosts the largest lion population among all African lion range countries. Our results add support that the species is structured into two lineages at the continental scale (West-Central *vs* East-Southern), underlining the importance of reviewing the taxonomic status of the African lion. Moreover, SNPs led to the identification of three lion clusters in Tanzania, whose geographical distributions are in the northern, southern and western regions. Furthermore, Tanzanian lion populations were shown to display good levels of genetic diversity with limited signs of inbreeding. However, their population sizes seem to have gradually decreased in recent decades. The highlighted Tanzanian African lion population genetic differentiation appears to have resulted from the combined effects of anthropogenic pressure and environmental/climatic factors, as further discussed.

## Introduction

The African continent still hosts a uniquely diversified megafaunal community [1]. This megafaunal diversity exceeds that of any other biogeographic region in the world [2]. For most savanna species, distinct subspecies are acknowledged at the African continental level. In recent years, the lion was proposed to be divided in two putative subspecies (*Panthera leo leo* for the Asian and West-Central African lion; *P*. *l*. *melanochaita* for the East-Southern African lion [3–7], supported by the SSC Cat Specialist Group (IUCN; P. Chardonnet, pers. comm.). This North/South dichotomy documented in other savanna mammals is believed to reflect common evolutionary responses to environmental changes mainly driven by major climatic oscillations that have occurred over the last 300,000 years [2]. However more recent specific micro-evolutionary changes associated with human activities (i.e. recent population fragmentation) were also shown to impact the genetic structure of African species at the population level. It has been estimated that the overall number of large mammals living in African protected areas decreased by 60% between 1970 and 2005, and by about 85% in West Africa over the same period [8]. The lion is no exception to this pattern. During the past two centuries, it has suffered major population decline and range contractions [9]. The population decline is, however, unequal within the lion distribution range [10–13]. The latest census in a sample of protected areas concluded a possible decrease of 62% between 1993 and 2014 within the West, Central and East African regions, while the Southern populations appeared to be more stable [14]. The long-term survival of lion populations in West and Central Africa is severely threatened with many more recent local extinctions noted, even within protected areas [13,15]. Following the IUCN Red List report, the species would currently only persist in a range of 1.65 million km^2^, which represents 8% of its ancestral distribution range [10,13,14,16].

Habitat loss, climate change, armed conflicts, illegal trade of lion body parts (e.g. for medicinal purposes), diseases and indiscriminate killing, primarily as a result of retaliatory or pre-emptive killing to protect human lives and livestock (‘human-lion conflicts’) are the main challenges threatening the species [17–20], as underlined in the IUCN Red List report [14]. Moreover, uncontrolled hunting and poaching of the lion’s wild prey, including medium to large-sized ungulates, is of major concern: these ungulate species are the target of bushmeat consumption, leading to collapses in the lion’s prey populations [21]. Direct competition for space and resources is also increasing with the steady expansion of crop-livestock farming [22]. Finally, as one of the African “Big Five”, the African lion is a major attraction for the hunting tourism industry. Trophy hunting carried out in a number of sub-Saharan African countries was shown to have a net positive impact in some areas, providing financial resources for the species conservation for both governments and local communities [14]. Nevertheless, it could also represent a threat to their survival if this activity is not well regulated and managed [18,23–25]. Therefore, with the increasing fragmentation of lion populations linked to anthropogenic pressures, disruption of the natural wildlife population admixture (i.e. gene flow) is expected to lead to genetic erosion [26,27].

Genetic tools can help gain further insight into the impact of population isolation on the long-term survival chances of threatened species. They can notably contribute to identifying management units or enable estimation of different demographic parameters, such as genetic differentiation, effective population size or inbreeding depression risks, which could help to draw up effective conservation practices for isolated and threatened populations. Indeed, isolated populations are more prone to inbreeding depression and extinction since low genetic diversity may lead to reduced fitness, while lowering the adaptive capacities of individuals to environmental change [28–30]. The consequences of inbreeding depression have previously been studied within different Felidae species, including lion populations from the Serengeti ecosystem, the Ngorongoro Crater and the Gir forest. The results highlighted a correlation between the population sizes, their genetic variability, testosterone level and counted sperm abnormalities [30]. Nevertheless, few fine-scale genetic studies have been conducted on this emblematic species [30–33], despite the fact that having an accurate idea of its ‘genetic health’ is essential for its conservation. With this concern, in 2006, the IUCN SSC Cat Specialist Group identified priority populations for conservation (“Lion Conservation Units” or LCUs). They represent ecological units of importance for lion conservation and were divided into three classes based on population size, prey base, threat level and habitat quality: class I for viable, class II for potentially viable and class III for significant but of doubtful viability [34]. However, LCUs do not yet take genetic parameters that measure a population’s genetic health for long-term conservation into consideration.

The objectives of the present study were therefore: I) to review the phylogeographical relationship and taxonomic status of African lion populations at the continental scale, and II) to study the genetic structure and diversity of the lion in Tanzania, the country with the largest lion population throughout its range. Tanzania currently includes five LCUs, almost all belonging to class I. This country is therefore of prime interest for lion conservation. In the present study, the following molecular markers were used: (i) a 1014 bp fragment of the mitochondrial cytochrome *b* gene, (ii) 11 microsatellites, and (iii) 9,103 single-nucleotide polymorphisms (SNPs), with the latter being obtained through the Genotyping-By-Sequencing (GBS) approach (Next-Generation Sequencing technology).

## Materials and methods

### Sampling

**Fig 1:**
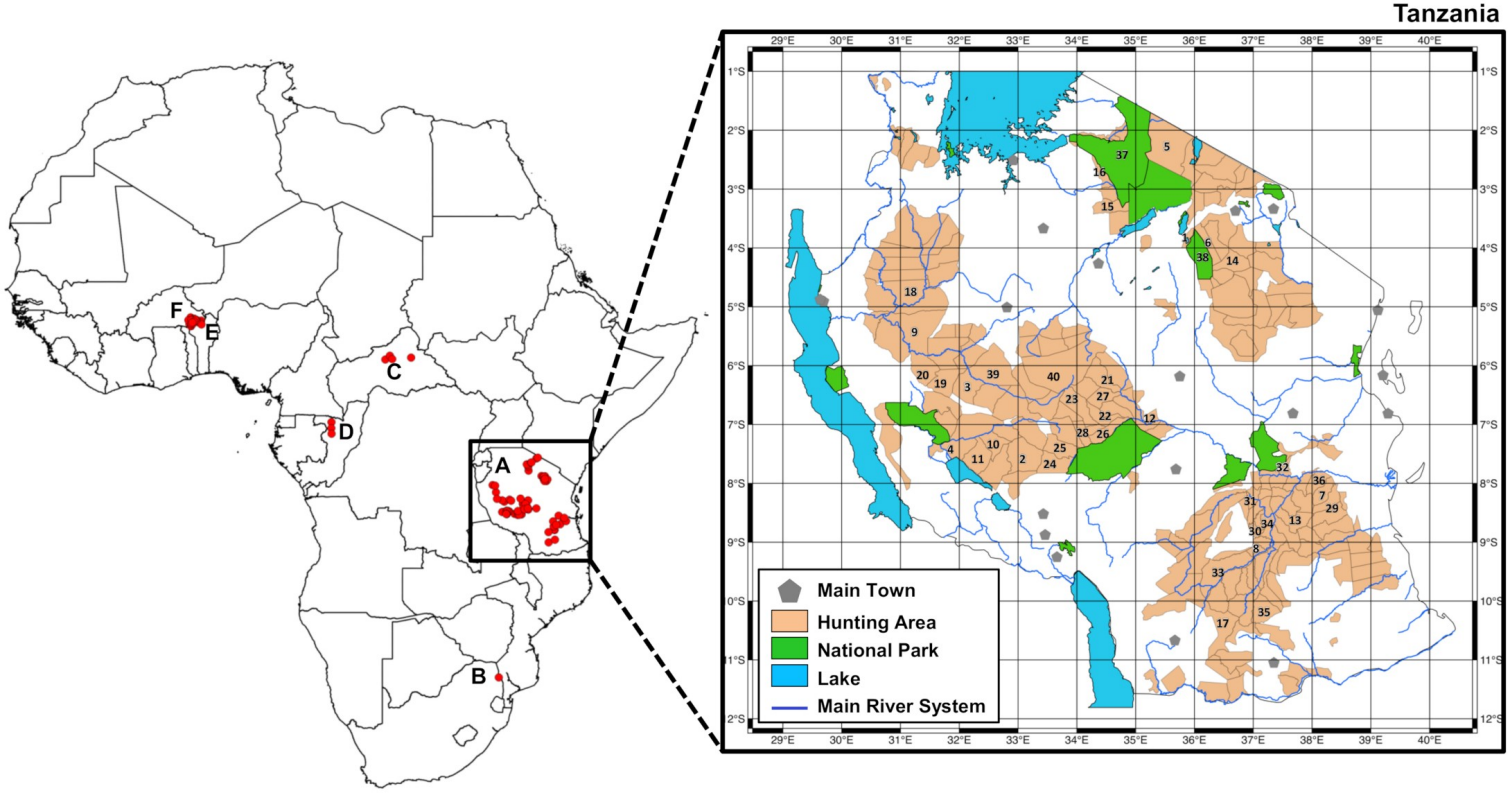
Map of Africa and detail of Tanzania showing sample locations. A: Tanzania; B: South Africa; C: Central African Republic; D: Congo; E: Benin; F: Burkina Faso. S1 Table includes specific information on the sample locations and their associated reference ID number as displayed on the present map.

The present study was conducted within the framework of the protocol agreement between the Wildlife Division of the Ministry of Natural Resources and Tourism of Tanzania and the IGF Foundation, in partnership with the Tanzania Wildlife Research Institute (TAWIRI). Our collection of samples from West-Central Africa and Tanzania was compiled by the Wildlife Division of Tanzania and the IGF Foundation under the auspices of the François Sommer Foundation (Fondation Internationale pour la Gestion de la Faune, France), and was supplemented with three samples from a breeding farm from South Africa provided by Mario Melletti (independent researcher, Italy) who had the required permits from the relevant national authorities. The samples were collected from dead animals, which were legally hunted following the rules of the Wildlife Division of the Ministry of Natural Resources and Tourism of Tanzania as well as the IGF foundation. Sampling was carried out in six countries, with larger scale sampling in Tanzanian protected areas: 74 from Tanzania, 20 from Burkina Faso, 1 from Benin, 3 from Congo, 3 from South Africa and 4 from the Central African Republic. A total of 105 tissue and hair samples, all from adult male lions (harvested from legally hunted specimens) were collected and stored in 96% ethanol. Sample details and locations are reported in S1 Table and Fig 1. Genomic DNA was extracted using the DNeasy Blood and Tissue Kit (QIAGEN Inc.) according to the manufacturer’s protocol. All DNA extracts were quantified using the Quant-iT^™^ Picogreen dsDNA Assay Kit (Fisher Scientific) and processed on a FilterMax^™^ F3 Multi-Mode Microplate Reader (Molecular Devices, LLC). To fulfill the GBS technic criteria (Cornell Core Facility genotyping guidelines), samples displaying less than 20 ng/μl DNA were concentrated using Amicon Ultra 0.5 mL Centrifugal Filters (Merck Millipore), according to the manufacturer’s instructions.

### Molecular markers

#### mtDNA cytochrome b gene

The cytochrome *b* (*cytb*) gene of the samples collected between 2005 and 2012 (N = 63; detailed sample information available on the Dryad Digital Repository: https://doi.org/10.5061/dryad.ff265) was amplified using forward L14724 (5’-CGAAGCTTGATATGAAAAACCATCGTTG) and reverse H15915 (5’-AACTGCAGTCATCTCCGGTTTACAAGAC) primers, targeting a 1140 bp fragment [35]. These samples covered the entire West-Central and Eastern studied areas. In order to recover the degraded material, four further internal specific primers were designed using BIOEDIT v7.1.11 and OLIGO 7 software by aligning *cytb* sequences from *P*. *leo* referenced on GenBank (GU131164-GU131185, AY781195-AY781210, DQ018993-DQ018996, DQ022291-DQ022301, AF384809-AF384818, KC495048-KC495058) [36,37]. The first primer pairs targeted a 680 bp fragment (PCytb-F1: 5’-ACATTCGAAATCACACCCCCTT; PCytb-R1: 5’-ATCTTTGATTGTATAGTATGGA) and the second, a 515 bp fragment (PCytb-F2: 5’-TCCATGAAACAGGATCTA; PCytb-R2: 5’-TAATGCCTGAGATGGGTA). The PCR reaction was carried out in a final volume of 25.5 μl, with each reaction containing 2.5 μl of DNA, 0.2μl of GoTaq DNA Polymerase (Promega), 5 μl of 5X GoTaq Reaction Buffer, 0.9 μl of each primer diluted at 10 μM, 0.8 μl dNTP at 10 μM, 0.7 μl BSA, 0.5 μl MgCl_2_ and 14 μl of Milli-Q water. Amplification was performed on a Thermal VWR UnoCycler with an initial activation step at 95°C for 15 min, followed by 35 denaturation cycles at 94°C for 40 s, annealing at 50°C for 45 s, and elongation at 72°C for 45 s, with a final extension at 72°C for 10 min. PCRs were resolved on an agarose gel and the positive products were sent to Macrogen Inc. for sequencing (both directions). Sequence Navigator v1.0.1 (Perkin-Elmer Applied Biosystem) and Chromaspro v1.7.5 (Technelysium Pty Ltd) software packages were used for electropherogram visualization, sequence correction, and primer trimming, whenever necessary, before alignment with ClustalW implemented in Bioedit v7.1.11 [36]. The database was then translated into amino acid sequences to verify that the coding region was free of stop codons and gaps.

#### Microsatellites

Eleven microsatellites, as presented in the study of Dubach *et al*. [38] addressing a genetic perspective on LCUs in East-Southern Africa, were selected (FCA014, FCA026, FCA030, FCA045, FCA077, FCA094, FCA096, FCA126, FCA132, FCA187 and FCA191 [39]). All the collected samples (2005 to 2015; N = 105) were genotyped (detailed sample information available on the Dryad Digital Repository: https://doi.org/10.5061/dryad.ff265). Four multiplex sets were designed based on size limitations and the amplification specificity (S2 Table). The PCR reactions were carried out in a final volume of 10 μl, containing 0.15 μl of each 10 μM diluted primer, 5 μl Multiplex Taq PCR Master Mix (QIAGEN) and 3–5 μl of DNA, depending of their initial concentration. PCR amplifications were performed in a Thermal VWR UnoCycler through an initial activation step (95°C for 15 min) followed by 35 cycles (denaturation at 94°C for 30 s, annealing at 57°C for 45 s, extension at 72°C for 45 s) and a final extension step at 72°C for 30 min. PCR products were genotyped on a 3130XL Genetic Analyzer using 2 μl of PCR product, 12 μl of Hi-Di^™^ formamide and 0.3 μl of GeneScan^™^-500 LIZ size standard (Applied Biosystem). Length variation determination was performed using GeneMapper v4.0 (Applied Biosystems).

#### Single-nucleotide polymorphism

**Preparation of the GBS library**: Since the generated microsatellite database did not provide sufficient resolution to answer our study question (see Result section), SNPs were investigated as an alternative. Of the 105 collected samples, only the ones meeting the Cornell Core Facility genotyping requirements (> 20 ng/μl DNA concentration, high DNA quality checked on agarose gel, according to Elshire *et al*. [40]), were retained for the SNP genotyping. After a preliminary RNase A digestion step (PureLink^™^ RNase A, 20 mg/ml, 2 h at room temperature), 50 μl of DNA of each of the 73 selected samples were prepared. The GBS library was constructed using the *Pst*I (CTGCAG) restriction enzyme. After digestion, adapters were ligated: barcode-containing adapters and common adapters. Following ligation, the samples were pooled, as described in Elshire *et al*. [40] and the library was sequenced on a Illumina HiSeq 2000/2500 (100 bp target length, single-end reads) by the Cornell University Life Sciences Core Facility (http://www.biotech.cornell.edu/brc/genomic-diversity-facility).

**DNA sequence alignment, SNP discovery and filtering**: The raw Illumina DNA sequence (hereafter called read) data were checked with FastQC [41] and processed through the GBS analysis v2 pipeline, as implemented in Tassel v5.2.15 [42] (http://www.maizegenetics.net/). The first step involved read trimming at 64 bp. Reads with unrecognized barcodes, present in less than 10 copies and less than 20 bp in size were discarded, as well as reads including a nucleotide position with a Phred quality score lower than the threshold of 20 (i.e. 99% of the base call accuracy). Moreover, reads over-represented in the database (more than 3 times the average sequencing depth) were also discarded. To determine copy numbers and genomic coordinates, sequence reads were aligned to the closely related *Felis catus* species reference genome using Bowtie v2.2.7 [43] with the very sensitive option (GenBank: ACBE00000000.1, GenBank assembly accession: GCA_000003115.1). The Tassel pipeline default parameters were used for filtering the resulting genotype table, except for the minimum minor allele frequency (mnMAF), which was set at 0.05. Third and fourth state alleles as well as indels were excluded. Further filtering of the putative SNP dataset was done to discard SNP genotypes present in less than 60% of the samples. Samples with more than 40% of missing data were also discarded. A python script was further used to remove all consecutive SNPs (Python v2.7.6) and to keep only one polymorphic site per read. SNPs physically found at read end positions were avoided because they were likely the result of sequencing errors as Illumina sequencing is more error prone with regard to read terminal positions [44]. The selection was performed using VCFtools v0.1.13 software [45]. Finally, we checked for outliers using BayeScan v2.1 software, with 100 prior odds [46]. Whenever necessary, Plink v1.07 software was used for random selection of SNP subsets [47].

### Statistical analyses

#### Preliminary requirements

Micro-Checker v2.2.3 was used to estimate the proportion of null alleles at each locus within our microsatellite dataset, as well as the stutter errors [48]. The markers were previously validated in the study of Dubach *et al*. [38] including samples covering a larger geographical area. This validation step was both performed on the entire database, as well as on the genotypes assigned to each cluster using Structure v2.3.4 (see Result section). Genotypes were then corrected relative to the results obtained with Micro-Checker v2.2.3 [48]. Tests for linkage disequilibrium (LD) between loci for each cluster, and the data were fit to the Hardy-Weinberg equilibrium (HWE) proportions for each locus separately and over all loci for each cluster using the Genepop web application (http://genepop.curtin.edu.au/; 1,000 dememorizations, 1,000 batches, 1,000 iterations per batch) [49]. Fisher’s method for combining independent test results across clusters and loci was used to determine the statistical significance of the test results.

Likewise for the SNP database, Arlequin v3.5 software was used to test genotypic distributions for conformance to HWE for each lineage and each cluster delineated with Structure v2.3.4 software (see Result section), where the significance was assessed using Fisher exact test *P*-values, and applying the Markov chain method (10,000 MCMC/10,000 dememorization steps) [50]. Loci would have been removed when out of equilibrium in more than one population. Whenever relevant, the *P*-value significance was sequentially Bonferroni-adjusted [51]. Genotypic LD was further tested using Plink v1.90b3.38 [47]. To predict the extent of linkage disequilibrium between each pair of loci, the r-squared statistic was chosen over the *D'* estimator. If a locus pair had an r^2^ value > 0.8 in multiple populations, the locus that was genotyped in the fewest individuals would have been removed.

#### Genetic structure analyses

Tree and network reconstruction details based on the *cytb* sequences, as well as compiled database from previous studies, are presented in supplementary S1 Document (section 1 in S1 Document). Bayesian clustering of microsatellites and SNP genotypes were performed using Structure v2.3.4, pooling individuals together independently of their spatial origin [52,53]. A burn-in of 100,000 iterations and 1,000,000 MCMC, and of 50,000 iterations and 100,000 MCMC, for each microsatellite and SNP dataset was applied, respectively. To cluster the samples, *K* from 1 to 5 and *K* from 1 to 10 were tested, with 10 iterations for each *K*, for each microsatellite and SNP dataset, respectively. The Markov chain convergence was checked between each 10 iterations for each *K*. The results and visual output of the 10 iterations for each *K* value were summarized using the web application CLUMPAK [54] (http://clumpak.tau.ac.il/index.html). The optimal number of clusters was assessed based on correction as defined by Evanno *et al*. [55]. The highest probability of each sample to belong to each cluster was used to determine its affiliation for the subsequent analyses. The analysis was run twice, the first time on the complete database and a second time on each group identified during the first run to check for finer-scale structure. In the present study, the ‘lineage’ term was used to describe the West-Central *vs* East-Southern axis structure (i.e. continental scale, as previously described in *P*. *leo* [5]) and the ‘population/cluster’ term was used to refer to the intra-lineage groups highlighted in the present study (i.e. local scale).

As an alternative approach to represent the genetic relationship among samples, a principal component analysis (PCA) and a neighbor-joining (NJ) tree (IBS distance matrix) were also performed using Tassel v5.2.15 on the SNP dataset [42]. The principal components indicated the directions with the most variance. Accordingly, the eigenvalues of all the principal components, the proportions of individual eigenvalues to the total variance (component contribution rates), and the raw scores of every sample for each of the principal components were calculated at continental and local scales. A final FCA was performed on the microsatellite database using Genetix v4.05 with default settings [56].

Finally, isolation by distance (IBD) patterns were determined by comparing pairwise F_ST_/(1- F_ST_) to the logarithm of the geographical distance (using the median of the distances between each individual and between each cluster identified) using the Isolation by Distance Web Service (IBDWS v3.23) (http://ibdws.sdsu.edu/~ibdws/) on the SNP dataset with 10,000 randomizations [57]. We then examined the decrease in genetic similarity over distance to assess the fine-scale genetic structure in Tanzania through a spatial autocorrelation analysis in GenAlEx v6.502 [58]. As this analysis is sensitive to missing data, the initial SNP database was reduced to 3,097 SNPs to allow a maximum of 9% missing data per sample. The spatial autocorrelation coefficient (*r*) was calculated among pairs of individuals (Multiple Dclass option). The autocorrelation coefficient and pairwise *r* values were divided into 18 distance classes (50 km each). These classes were chosen arbitrarily since the home range size of the African lion varies considerably between seasons and across study areas (e.g. 20–45 km^2^ in Manyara NP and Ngorongoro Crater (Tanzania) [59–61], to more than 2,000 km^2^ in arid ecosystems such as Etosha NP (Namibia) [62]). The seasonal home range size was suggested to be strongly linked to the pride biomass [63]. Additionally, the prey abundance and distribution, as well as interactions with conspecifics and intraspecific competition for space, would also influence home range sizes [63]. A null distribution of *r* values for each distance class was obtained by 9999 permutations and the confidence intervals for *r* were estimated by 9999 bootstraps with replacement, and plotted in a correlogram [58]. The extent of the detectable spatial genetic structure was approximated as the distance class at which *r* was no longer significant and the intercept crossed the x-axis.

#### Genetic diversity and population differentiation

*F*-statistics (*F*_*ST*_, *F*_*IS*_), allelic richness (*A*_*R*_) and heterozygosities (*H*_*E*_, *H*_*O*_) were investigated based on our microsatellite dataset, using Genetix v4.05 software [56]. The number of alleles (*N*_*A*_) and private alleles (*P*_*A*_) per population were estimated using Genepop v4.2 [49]. Further details about the *cytb* genetic diversities analyses and results can be found in supplementary document S7 (section S7.2).

For the following analyses, summary statistics were estimated only for lineages (continental scale) and clusters (population scale) including more than 5 individuals, on both the complete SNP database and a reduced subset of 3,097 SNPs (maximum of 9% missing data accepted). The genetic diversity of each group was assessed by calculating the expected (*H*_*E*_) and observed (*H*_*O*_) heterozygosities, the fixation indices and the inbreeding coefficient (*F*_*IS*_), using GenAlEx v6.502 and Arlequin v3.5 [50,58], with 1,000 permutations for significance. An exact test of population differentiation of pairwise weighted mean *F*_*ST*_ [64] was performed using the same software (10,000 permutations for significance, with an allowed missing data level of 0.05). A visual *F*_*ST*_ heatmap was reconstructed with RStudio v3.3.1 software using the heatmap.plus package v2.18.0 (https://github.com/alexploner/Heatplus) [65].

The hierarchical distribution of genetic variance among and within populations was assessed using an analysis of molecular variance (AMOVA) performed with Arlequin v3.5 on the complete SNP dataset [50]. The AMOVA analysis was partitioned into covariance components to calculate the variance among clusters relative to the total variance (*F*_*CT*_), the variance among populations within clusters (*F*_*SC*_) and the variance among populations relative to the total variance (*F*_*ST*_) (1,000 permutations for significance). The populations and groups for the AMOVA analysis were defined according to the clustering results obtained with Structure v2.3.4 [52]. Finally, recent demographic bottlenecks were further investigated with Bottleneck v1.2.02 [66], computing the average heterozygosity which is compared to the observed heterozygosity to determine if a locus expresses a heterozygosity excess/deficit [66]. Estimations were based on 1,000 replications, repeated 10 times on database subsets of about 250 randomly selected SNPs for each identified cluster. Mode shifts in allelic frequency distribution were further assessed using the same software.

## Results

### Molecular markers

#### mtDNA cytochrome b gene

In addition to the 74 sequences from GenBank, 54 samples were newly sequenced for the *cytb* gene. Three samples (BUR10, CON2 and CON3) failed at providing positive amplification results. Six other samples (TAZ10, TAZ18, TAZ41, TAZ43, TAZ44 and TAZ46) could only be partly amplified using the internal primers, and were therefore discarded from the following analyses. Once aligned, the total overlapping fragment size was of 1014 bp. Seventeen haplotypes were identified, including one new haplotype (S3 Table- GenBank accession numbers: MG677918- MG677922). Of these 1014 bp, 32 sites were variable, 17 were parsimoniously informative and 15 were singleton-variable sites. The transition/transversion rate ratios for k1 was 15.54 (purines) and for k2 was 15.91 (pyrimidines). The nucleotide frequencies were 27.6, 29.6, 14.5 and 28.2 for A, C, G and T, respectively.

#### Microsatellite and single-nucleotide-polymorphism genotypes

The 11 microsatellites genotype database included 73 samples from Tanzania, 3 from South Africa, 2 from Congo, 4 from the Central African Republic (CAR), 20 from Burkina Faso and 1 from Benin (N_TOTAL_ = 103, Data available from the Dryad Digital Repository: https://doi.org/10.5061/dryad.ff265). Samples TAZ57 from Tanzania and CON1 from Congo failed at providing genotypes. Micro-Checker v2.2.3 allowed us to identify the presence of null alleles among 5 microsatellites, which were corrected accordingly [48]. The Hardy-Weinberg exact test (Genepop v4.2) performed at each locus separately and over all loci for each cluster showed no deviation from the expected frequencies after Bonferroni’s correction (*p*-value > 0.05). Moreover, no linkage disequilibrium (LD) was observed among the microsatellite markers used (*p*-value > 0.05).

Concerning the single-nucleotide-polymorphism (SNP) identification pipeline, among the 73 samples initially included (Tanzania, Burkina Faso, Benin and CAR), 66 passed the filtering criteria and were kept for the following analyses. The first filtering steps performed with Tassel v5.2.15 allowed the selection of 270,814 reads out of the 206,203,194, that were both correctly barcoded and of good quality (Phred score). Of these reads, 65% aligned to the *Felis catus* genome at one position (21.22% aligned at multiple positions, 13.77% did not align). The total number of polymorphic sites identified from these 176,029 reads aligning at one position was of 66,033. This number decreased to 23,138 SNPs when filtering for missing data. Moreover, by retaining only one polymorphic site per read, the number decreased to 9,114 SNPs. Finally, the 11 outlier SNPs identified with BayeScan v2.1 were also discarded from the database (Data available from the Dryad Digital Repository: https://doi.org/10.5061/dryad.ff265). The T_S/_T_V_ ratio was of 2.83, while ratios substantially less than 2 can be indicative of sequencing errors.

All populations identified with the Structure v2.3.4 were shown to be in HWE. Some SNPs were found to be in LD within one of the Tanzanian populations. These were not removed because the same SNPs were not in disequilibrium within the other identified populations.

### Statistical analyses

#### Genetic population structure

**Fig 2:**
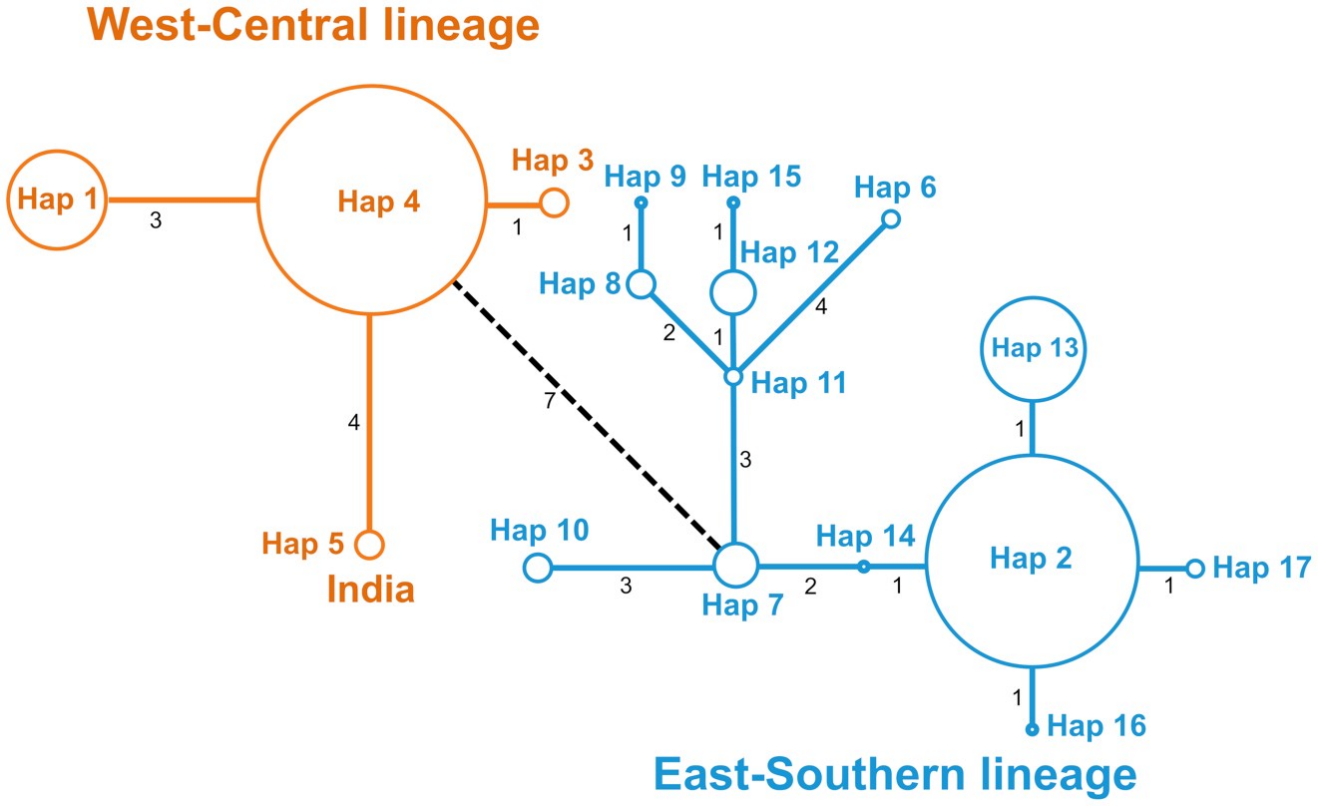
Minimum spanning network reconstruction of *P*. *leo* showing genetic relationships among the 17 *cytb* haplotypes. The size of circles is proportional to haplotype frequency. The number of mutational steps separating the haplotypes is indicated on the connecting branches in black. Orange: West-Central lineage (including India), blue: East-Southern lineage.

**Continental scale**: All molecular markers led to the identification of a partitioning into two supported lineages at the continental scale. The lineages were separated by 7 mutational steps on the minimum spanning network (Fig 2), and supported by a bootstrap (BS) of 1000 on the ML tree reconstruction (S1 Fig). The West-Central lineage included the 4 Indian samples from the Gir Forest, which were separated by 4 mutational steps from the African individuals (Fig 2). Two haplotypes (hap2 and hap4) appeared to be prevalent in frequency but this may represent an artefact linked to the sampling that was more extended for some localities (S3 Table). In general, adjoining countries shared the same haplotypes, but some exceptions appeared (S3 Table). Hap6, 8, 9, 11, 12 and 15 were clustering together and were specific to Southern Africa, while hap7 and 10 were only found in East Africa. The other haplotypes from this lineage were shared between East and Southern Africa. Likewise, hap4 was shared between West and Central Africa, while hap1 only occurred in West Africa and hap3 was only found in Central Africa. The ML tree showed the same pattern, although not all branches were supported with high BS (S1 Fig).

**Fig 3:**
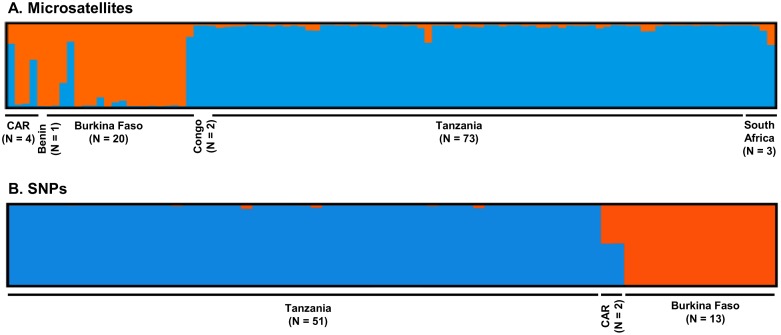
*P*. *leo* lineages in Africa inferred with Structure v2.3.4 software based on the A. microsatellite and B. SNP dataset, after Evanno *et al*. [55] correction (*K* = 2) (CLUMPAK). The lineage membership of each sample is shown by the color composition of the vertical lines, with the length of each color being proportional to the estimated membership coefficient. CAR: Central African Republic, orange: West-Central lineage, blue: East-Southern lineage.

**Fig 4:**
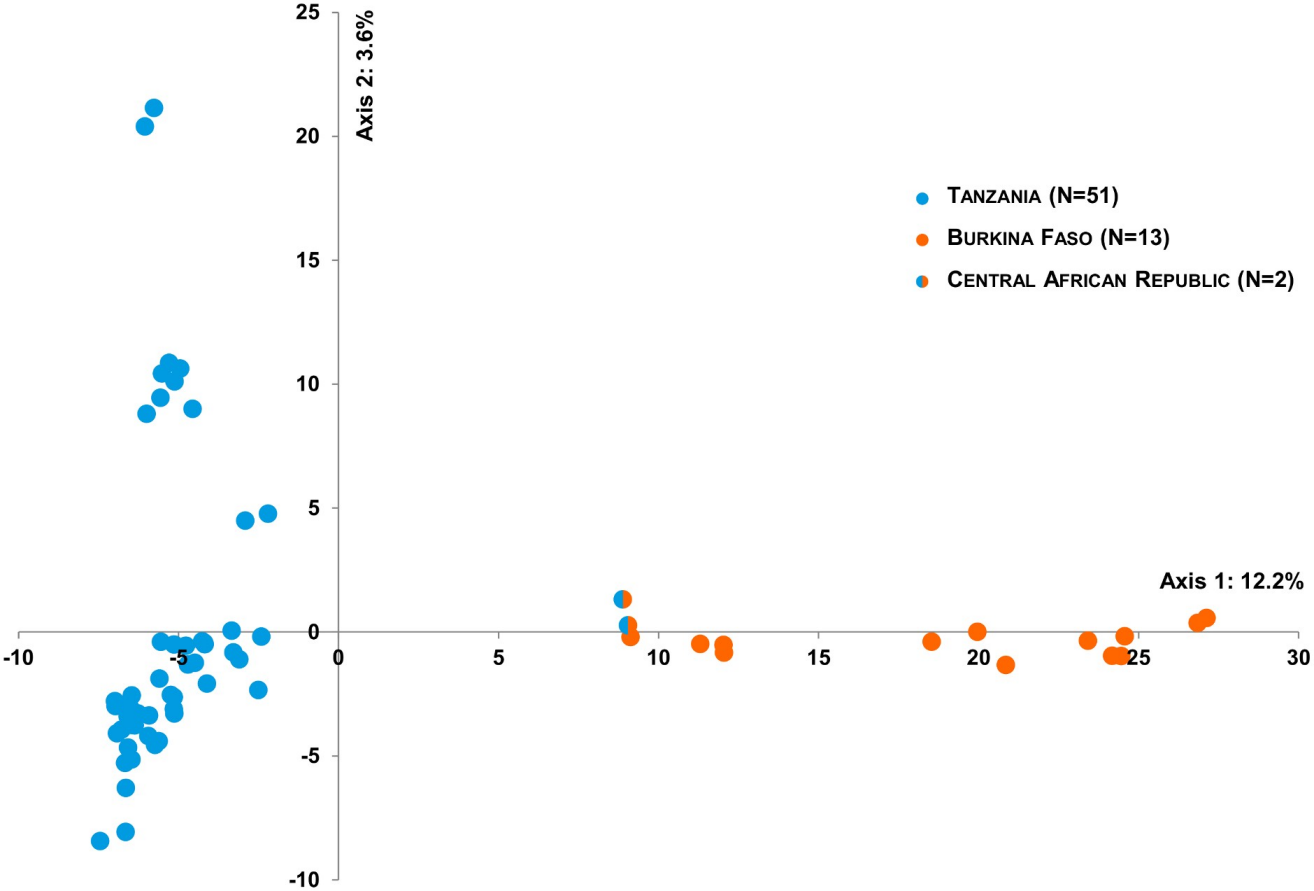
Principal component analysis (PCA) performed with Tassel v5.2.15 on the whole SNP dataset at the continental scale (N = 66). Orange: Burkina Faso samples (West-Central lineage), blue: Tanzanian samples (East-Southern lineage). The two samples from CAR harbor both colors, indicative of an intermediate genetic composition (see Fig 3B).

The Structure v2.3.4 analyses also indicated the existence of two lineages at the continental scale, based on both microsatellite (N = 11/103 samples) and SNP (N = 9,103/66 samples) datasets. The results were interpreted using the *ΔK* method, as described by Evanno *et al*. [55]. The highest recorded *ΔK* was for *K* = 2 (Fig 3, Figure A in S2 file and Figure A in file S7). The signature was clearer based on SNPs than on microsatellites. In the Central African Republic for example, all the samples genotyped with SNP markers (N = 2; RCA2 and RCA4) appeared to be admixed (Fig 3B). Based on microsatellites, two of the four samples (RCA1 and RCA4) included in the analysis appeared to be admixed (Fig 3A). Also, the samples from Burkina Faso (3 of 20 samples) appeared to be admixed based on microsatellites (BUR3, BUR4 and BUR20; Fig 3A), while it wasn’t the case based on SNPs (Fig 3B). However, SNP genotypes were available for BUR20 but not for BUR3 and BUR4 due to DNA quality issues. Therefore, direct comparisons between clustering results and molecular markers should be taken with caution. This was further supported by the FCA performed on the microsatellite dataset (three axes explaining 17.53% of the genetic variability)), and the PCA performed on the SNP dataset (first two PC explaining 15.7% of the genetic variability) (S3 Fig and Fig 4).

**Fig 5:**
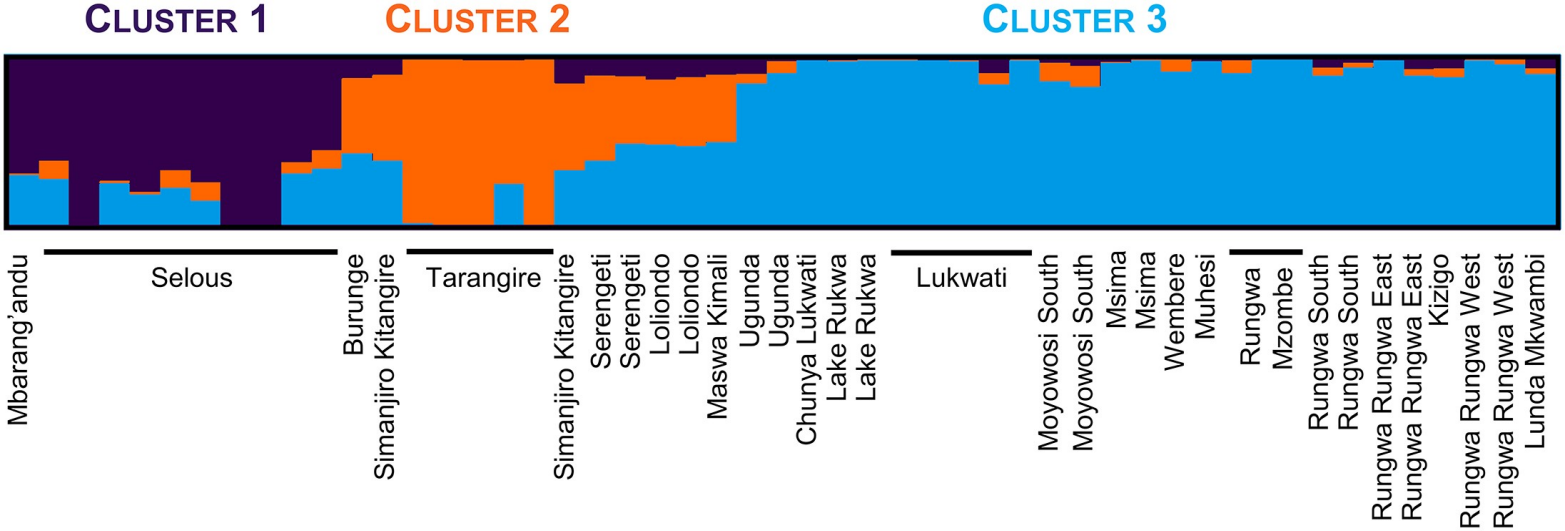
Tanzanian clusters inferred with Structure v2.3.4 software based on the SNP database, after Evanno *et al*. [55] correction (*K* = 3) (CLUMPAK). The cluster membership of each sample is shown by the color composition of the vertical lines, with the length of each color being proportional to the estimated membership coefficient. A spatial representation is shown in Fig 7.

**Fig 6:**
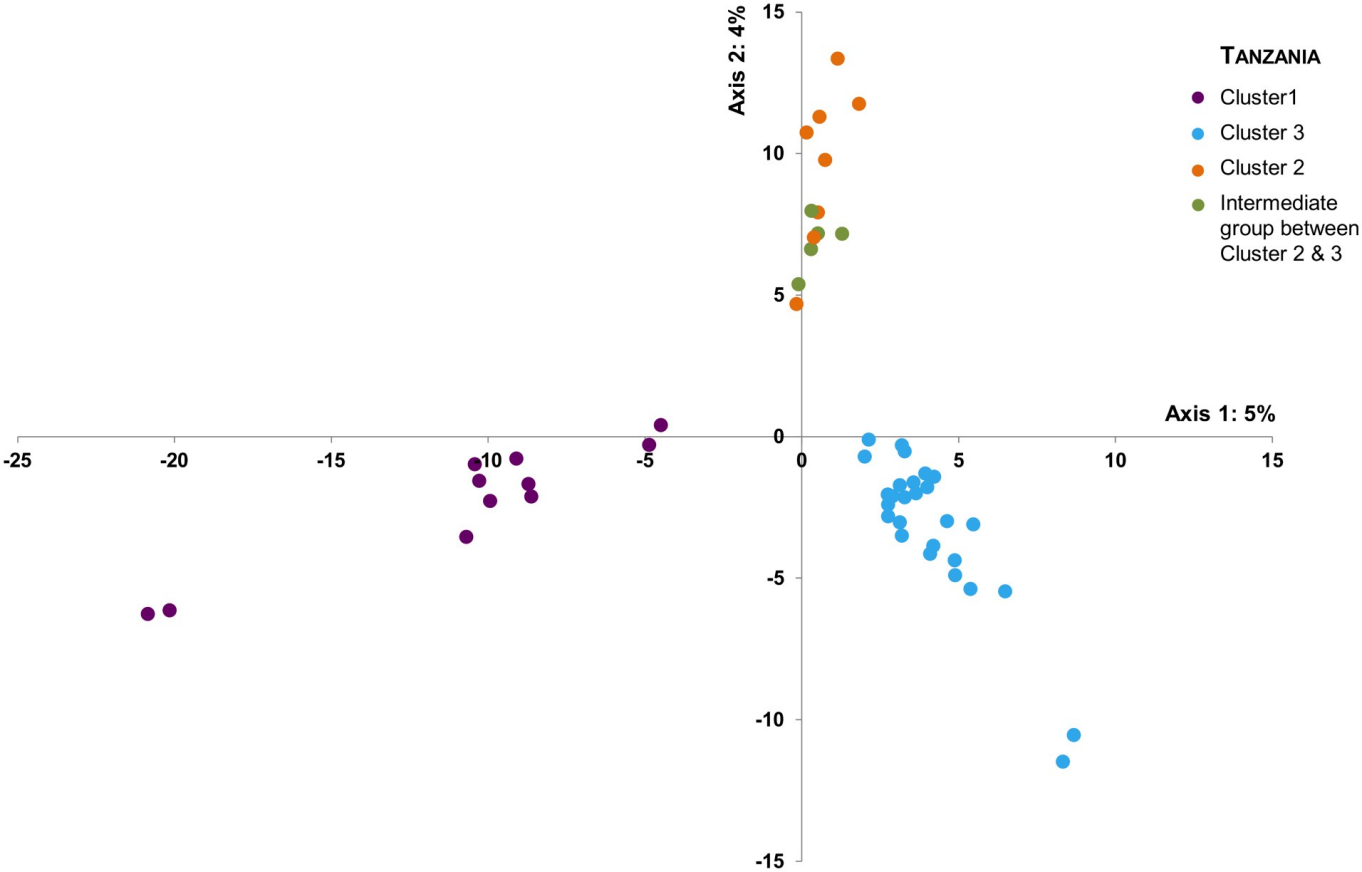
Principal component analysis (PCA) performed with Tassel v5.2.15 on the SNP dataset including all samples from Tanzania (East-Southern lineage).

**Fig 7:**
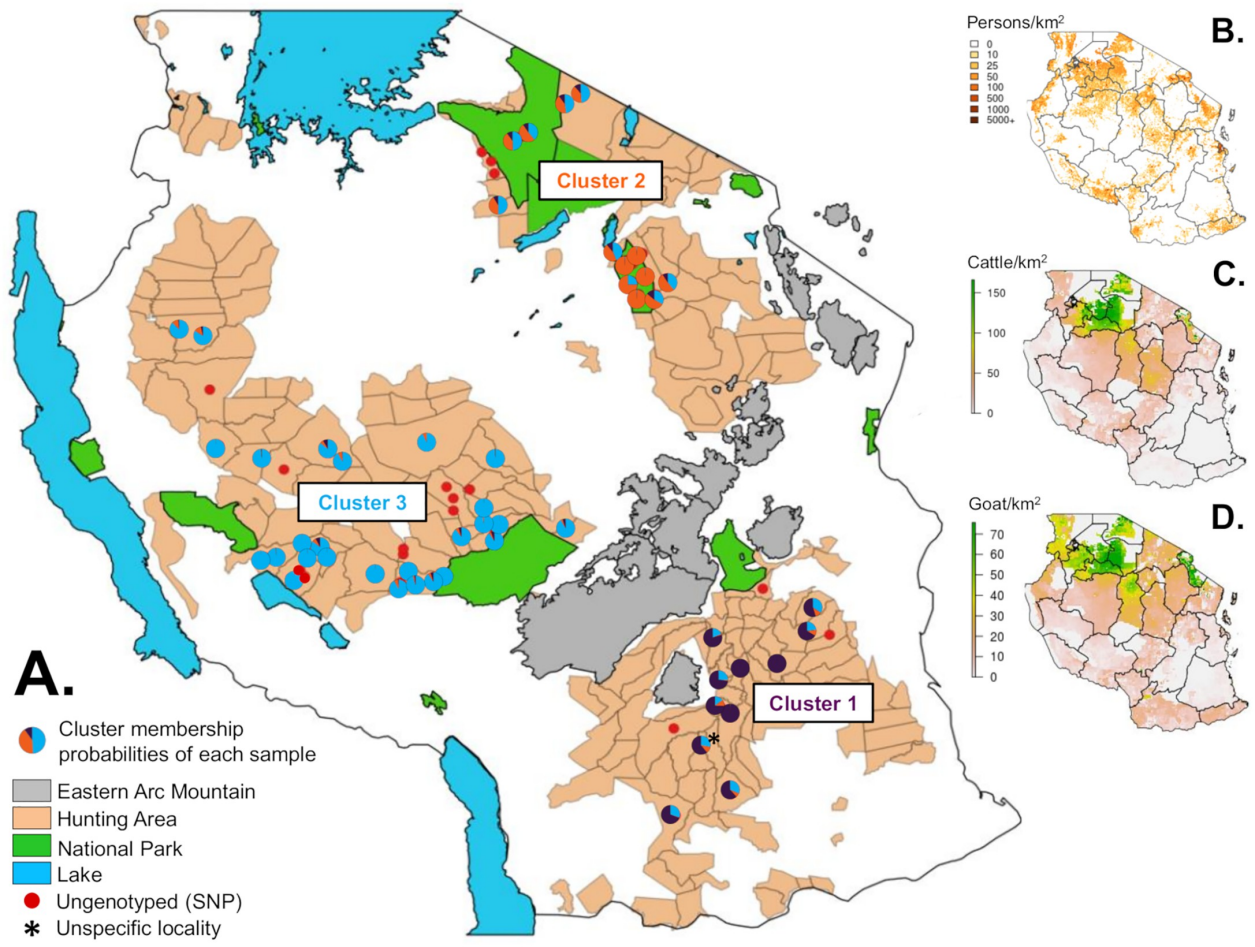
Maps of Tanzania displaying. A. the clustering analysis results based on our SNP database (each pie chart (dot) represents one individual, colors of the pie chart represent each individual’s assignment probabilities to each of the three clusters), B. the human population density in 2015 (http://www.worldpop.org.uk/; CC BY 4.0), C. the spatial distribution (head per km^2^) of cattle, and D. of goats in Tanzania (https://geonetwork-opensource.org/; open source software), the three later maps reprinted and adapted from http://gfc.ucdavis.edu/profiles/rst/tza.html under a CC BY 4.0 license [67].

**Tanzanian scale**: At the Tanzania country scale, clustering analyses (Structure and FCA) based on microsatellites did not identify a finer-scale structure (*K* = 1) (S7B Fig). Based on SNPs, three populations emerged from the Structure analysis (Fig 5 and Figure B in S2 file). The populations were also identifiable on the NJ tree (S6 Fig) and on the PCA (Fig 6). These populations were geographically structured among the South (Cluster 1), the North (Cluster 2) and the Western (Cluster 3) regions of Tanzania (Fig 7- with each pie chart representing one individual and its respective membership probabilities for each of the three Clusters). However, it is interesting to note that some individuals displayed intermediate probabilities of belonging to either Cluster 1 and Cluster 3, or Cluster 2 and Cluster 3. (Fig 5- with each sample membership coefficients displayed as vertical lines). A clear cut-off delineating each three Tanzanian Clusters was not evident from the results.

A statistically significant isolation by distance (IBD) pattern in Tanzania was not found based on our database of 3,097 SNPs (*r*  =  -0.071, *p*   =  0.66) among our sampling locations. Spatial autocorrelation showed a pattern of decreasing relatedness with increasing distance. Autocorrelation decreased to a level not significantly different from 0 to about 750 km (S4 Fig). Nevertheless, only two samples presented a physical separation of more than 750 km in distance, which we consider as no longer representative and would rather indicate an absence of isolation by distance.

#### Genetic population diversity and differentiation

**Continental scale**: Based on the 11 microsatellites, moderate *F*_*ST*_ (0.144) and *G*_*ST*_ (0.089) were highlighted between the two main lineages. The analysis of molecular variance indicated that most of the variation occurred within lineages (85.6%), as compared to those observed among lineages (14.4%). Inbreeding coefficient estimates showed more pronounced homozygote excess within the West-Central lineage (*F*_*IS*_ = 0.138) as compared to the East-Southern lineage (*F*_*IS*_ = 0.064) (Table 1). Likewise, the allelic richness and the number of private alleles were higher on the East-Southern lineage (*A*_*R*_ = 7.27—*P*_*A*_ = 32) as compared to the West-Central lineage (*A*_*R*_ = 5.09—*P*_*A*_ = 8), but this may be an artefact associated with the different sampling sizes for each lineage (Table 1). Based on the SNP dataset, a moderate *F*_*ST*_ (0.271) between both main lineages was recorded. According to the previous results, the number of private alleles based on the SNP dataset was also higher for the East-Southern lineage (*P*_*A*_ = 790) as compared to the West-Central lineage (*P*_*A*_ = 182) (Table 1).

**Table 1:** Descriptive statistics of the genetic diversity within each lineage and each Tanzanian cluster based on 11 microsatellites and 3,097 SNPs (maximum missing data of 9%), calculated with Genetix v4.05 (*A*_*R*_, *H*_*O*_, *H*_*NB*_, *F*_*IS*_) and Genepop v4.2 (*N*_*A*_, *P*_*A*_) for microsatellites, and with GenALex for SNPs.

	*N*	*N*_*a*_	*P*_*a*_	*A*_*R*_	*H*_*O*_	*H*_*NB*_	*F*_*IS*_
**Microsatellite—Lineages**							
West-Central	21	56	8	5.09	0.56 ± 0.15	0.66 ± 0.13	0.14 ± 0.03
East-Southern	78	80	32	7.27	0.68 ± 0.09	0.73 ± 0.08	0.06 ± 0.02
**SNP—Lineages**							
West-Central	15	/	182	/	0.19 ± 0.01	0.26 ± 0.01	0.23 ± 0.01
East-Southern	51	/	790	/	0.24 ± 0.01	0.30 ± 0.01	0.19 ± 0.01
**SNP—Clusters**							
TAZ Cluster 1	11	/	/	/	0.239 ± 0.004	0.275 ± 0.003	0.078 ± 0.007
TAZ Cluster 2	9	/	/	/	0.203 ±0.003	0.285 ± 0.003	0.210 ± 0.007
TAZ Cluster 3	31	/	/	/	0.257 ± 0.003	0.299 ± 0.003	0.124 ± 0.005
Burkina Faso	13	/	/	/	0.165 ± 0.003	0.240 ± 0.004	0.242 ± 0.007

*N*: number of samples, *N*_*A*_: number of alleles, *P*_*A*_: number of private alleles, *A*_*R*_: number of alleles per locus, *H*_*O*_: observed heterozygosity, *H*_*NB*_: unbiased expected heterozygosity, *F*_*IS*_: inbreeding coefficient. As the CAR was represented by a very small sampling size, summary statistics and demographic parameters were not estimated for the latter.

**Fig 8:**
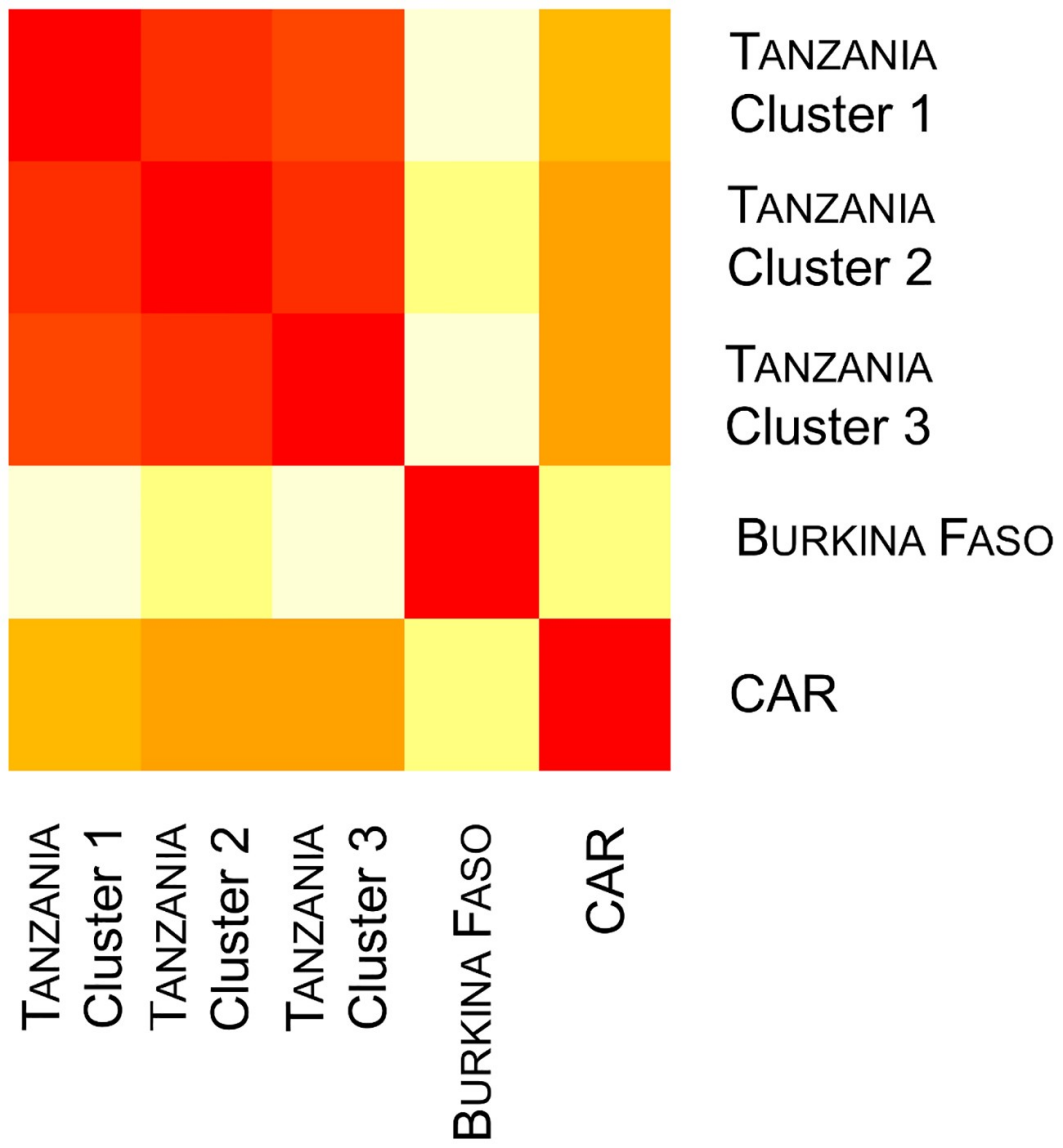
Heatmap representation displaying an increasing color intensity between less differentiated clusters, obtained based on the SNP dataset.

The AMOVA analysis performed on the SNP dataset highlighted a higher level of genetic variation within clusters (75.35%) than among lineages (18.35%), with the lowest variation occurring among clusters within both lineages (6.31%) (Table 2). Regarding the pairwise *F*_*ST*_ estimates, all were significant (*p*-values < 0.05) (Table 3). Both the *F*_*ST*_ value and the heatmap representation (Fig 8) indicated that geographically closer clusters were less differentiated.

**Table 2:** AMOVA results performed on the SNP dataset including clusters with more than 5 samples (Arlequin v3.5).

variation Type	d.f.	Sum of squares	Component Variance	% of variation	fixation indices	P-value
**Among lineages**	1	3098.39	52.87	18.35	*F*_*CT*_ = 0.183	0.089 ± 0.010
**Among clusters within lineage**	4	2125.87	18.17	6.31	*F*_*SC*_ = 0.077	0.000 ± 0.000
**Within Clusters**	126	27357.84	217.13	75.35	*F*_*ST*_ = 0.247	0.000 ± 0.000
**Total**	131	32582.098	288.171			

**Table 3:** Pairwise *F*_*ST*_ between each identified cluster using SNP markers.

	**TAZ** **Cluster 1**	**TAZ** **Cluster 2**	**TAZ** **Cluster 3**	**Burkina Faso**	**CAR**
**TAZ** **Cluster 1**	0	***	***	***	**
**TAZ** **Cluster 2**	0.083	0	***	***	*
**TAZ** **Cluster 3**	0.057	0.046	0	***	***
**Burkina Faso**	0.286	0.282	0.246	0	**
**CAR**	0.193	0.178	0.160	0.220	0

(*) *p*-value < 0.05,

(**) *p*-value < 0.01 et

(***) *p*-value < 0.001.

**Tanzanian scale**: Lower *F*_*ST*_ were found among Tanzanian clusters, with the highest value observed between Cluster 1 (South region of Tanzania) and the other two (Table 3). The observed heterozygosity within clusters was lower than the unbiased expected heterozygosity, which would be indicative of an excess of homozygotes. Inbreeding coefficient (*F*_*IS*_) estimates indicated more pronounced homozygote excess in Cluster 2 (North region of Tanzania—*F*_*IS*_ = 0.210) compared to the two other identified clusters (Table 1). Finally, to determine whether the Tanzanian clusters had undergone recent demographic contraction, excess in heterozygosity at mutation-drift equilibrium (*H*_eq_) was investigated using Bottleneck. The results indicated that all three Tanzanian clusters showed significant (*p* < 0.05) heterozygosity excess under all mutation models (IAM and SMM), as well as a mode-shift.

## Discussion

### Continental scale genetic structure

At the continental scale, all molecular markers (*cytb* gene, microsatellites and SNPs) supported the existence of two main lineages (West-Central Africa and India (i.e. Gir Forest) *vs* East-Southern Africa) for the African lion (*Panthera leo leo*), as highlighted by previous studies [3–7]. The more extensive sampling allowed to identify a new *cytb* haplotype in Tanzania, while the geographical distribution of four other haplotypes was extended to newly sampled areas. This main continental-scale division is a pattern that has also been observed in many other savanna mammals and corresponds to common evolutionary responses to environmental changes that drove their genetic differentiation over time [2]. High differentiation, as indicated by the high *F*_*ST*_ of the *cytb* gene (supplementary document S7), is usually observed between subspecies [68]. Our results therefore supported previous propositions encouraging a taxonomic revision of this species, with a separation between the Western-Central (including Asian lion) and East-Southern populations, as distinct subspecies or at least as distinct management units (MUs [69]).

### Tanzanian scale genetic population structure and differentiation

While based on the present microsatellite set, no finer scale structure within Tanzania (insufficient resolution) could be identified, SNPs enabled the identification of three clusters, geographically distributed across the country among the South (Cluster 1), North (Cluster 2) and Western (Cluster 3) regions. These specific microsatellites are therefore not recommended for fine scale studies. The higher number of SNP markers used in the present study in comparison to microsatellites seems to be the best explanation for the observed differences in our results, especially considering the present sampling number and coverage. However, previous studies revealed that four to twelve times more SNPs are needed for population structure inference to match the statistical power of one microsatellite [70]. Following this assumption, the number of SNPs included in this study would, in the worst case, be equivalent to the use of hundreds of microsatellites.

The population structure highlighted on the basis of SNPs did not seem to result from an isolation-by-distance process (IBD and spatial autocorrelation analyses). Therefore, the differentiation was probably linked to the combined effects of both anthropogenic pressure and environmental/climatic factors. Indeed, the presence of the Eastern Arc Mountains chain associated with the land-use pattern may represent a major biogeographical barrier to lion dispersal. This chain of mountains runs from northeastern to southwestern Tanzania and is geographically situated between Cluster 1 and the two other identified clusters [71], while Cluster 1 also had the highest pairwise *F*_*ST*_ estimates (Fig 7). A similar population genetic structure has been reported in the sable antelope (*Hipotragus niger*), with long-term isolation of distinct lineages in Western and Southern (i.e. Selous GR) Tanzania, each on either side of the Eastern Arc Mountains ([72,73], P. Vaz Pinto, pers. comm., ongoing research based on mitochondrial DNA sequences and microsatellites). On the other hand, the three identified clusters appeared to be geographically separated by corridors of agropastoral lands, associated with high human and livestock densities (Fig 7) (almost half of the country’s surface area is allocated to agricultural activities (FAO 2013- http://gfc.ucdavis.edu/profiles/rst/tza.html)). For example, the Kilombero valley between Clusters 1 and 3 is characterized by large cash crop plantations, with many villages and roads. Several studies have highlighted that carnivores tend to avoid regions with high human activity, even though avoidance is not total [74–77]. Indeed, large carnivore requirements often conflict with those of local people relying on farming and livestock husbandry [20]. The African lion is often the first of the large carnivore species to be actively persecuted when living alongside communities and livestock [78,79]. Behavioral changes in response to human-caused mortality risk were highlighted in lions in response to land use [74]. These corridors of agropastoral lands, associated with environmental barriers such as the Eastern Arc Mountains, are therefore believed to act as main dispersion barriers among the identified clusters and may well have led to the observed differentiation, even though no accurate information are available on the historical conservation status (range, population size, threats) of the lion in Tanzania. The differentiation level between the three Tanzanian populations was estimated to be low to moderate, as highlighted by the pairwise *F*_*ST*_ indices (Table 3), with the highest values obtained between Clusters 1 and 2 (*F*_*ST*_ = 0.085) and the lowest between Clusters 1 and 3 (*F*_*ST*_ = 0.046), suggesting relatively recent differentiation as further discussed hereunder.

Among the three identified clusters, some samples displayed an admixed genetic pattern and could not be clearly assigned to one cluster. For instance in the Northern region of Tanzania, genetically admixed samples between Clusters 2 and 3 were clearly identified (Fig 7). These samples came from the areas of Serengeti, Loliondo, Burunge and Maswa Kimali, which were geographically closer to the Northern cluster (Cluster 2), but were genetically assigned to Cluster 3 (Western Tanzania) although with low posterior probabilities (close to 0.5 in Fig 5 and highlighted in green in Fig 6). Different hypotheses could explain the highlighted intermediate pattern. First, it may have resulted from admixtures through recent gene flow between these two clusters since no physical barriers delimited the lion strongholds in Tanzania. Nevertheless, even if males have dispersal capabilities, the distance between North and Western Tanzania is about 400 km, while the Central Tanzanian regions are generally characterized by high human densities (Fig 7), thus hampering movements between lion strongholds and providing low support to this hypothesis. It may also be linked to a sampling bias since only a few individuals were concerned, possibly reaching the limits of the assignment capacities of the clustering software. Nevertheless, all individuals with an intermediate genetic composition were geographically sampled within neighboring areas and not randomly dispersed, thus indicating geographic consistency. Moreover, the same pattern was revealed within the PCA and NJ tree (Figs 6 and 7). Therefore, a sampling bias does not seem to explain the present results. However, the shared genetic material may also reflect an ancient connectivity between the two clusters (past panmictic lion population), with the time elapsed since the differentiation not being long enough to observe complete cluster sorting. This seems to be the most supported hypothesis in regard of our results.

### Tanzanian population genetic diversity

Inbreeding depression risks were investigated in each of the three identified clusters based on the *F*_*IS*_ index. The lowest estimate was obtained for Cluster 1 (*F*_*IS*_ = 0.078) located in Southern Tanzania, indicating a low risk of inbreeding depression. A recent census of the lion population in a sample of 1,300,000 ha in the Selous GR (26% of the overall protected area), which is devoid of human and cattle populations, confirmed that the lion density was still substantial; the Selous GR has therefore been proposed as an important lion stronghold in Tanzania (Wildlife Division, unpublished data). An intermediate *F*_*IS*_ was obtained for Cluster 3 (*F*_*IS*_ = 0.124), with the highest value recorded for Cluster 2 (*F*_*IS*_ = 0.210) located in the North of the country. While the lion density in the Selous GR was shown to be homogenous throughout the studied area (4.2 lions/100 km^2^ (2.6–5.2)- Wildlife Division, unpublished data), the ecosystem was more heterogeneous in the Northern region, with lion densities markedly fluctuating between sites (reviewed in [80]). The inbreeding coefficient may reflect this particularity, as the Northern lion population may suffer from higher human pressure in the Serengeti/Tarangire ecosystems, associated with higher livestock densities in the areas surrounding the national parks (Fig 7). Moreover, the Cluster 2 *F*_*IS*_ was similar to those obtained for the Burkina Faso lion population (*F*_*IS*_ = 0.242 –samples from Pagou-Tandougou, Singou, Kourtiagou, Koakrana, Konkombouri and Pama). Given that the Western African lion population has undergone a more serious population size decrease recently (see Introduction), these similar values suggest that Cluster 2 is characterized by an increased risk of inbreeding depression, although not yet alarming.

### Conservation implications

With the increasing human encroachment in landscapes, wildlife habitat fragmentation may become a serious issue for the long-term survival of wild species that barely co-exist with humans. In 2015, Tanzania had an annual human population growth rate of 3.1% (http://data.worldbank.org/), indicating that human pressure on wildlife habitats is not expected to decrease in the short-term. The present results may directly impact the management practices of this emblematic species in Tanzania, a major lion stronghold. In order to maintain historical levels of genetic variability in the long term, genetic admixture among recently-diverged clusters could be necessary through the establishment of ecological corridors or the translocation of individuals. Nevertheless, species-specific constraints could lead to failure of such programs. In the case of translocations, for example, the species-specific social structure (female philopatric behavior, fission-fusion pattern, male competition, etc.) could seriously complicate the inclusion of translocated individuals into new prides [81], and therefore the reproductive success would likely be challenged [82]. When management actions are undertaken, it was shown that behavioral responses of monitored translocated individuals do not always conform to the manager expectations [83].

The uninterrupted past connectivity between lion populations (panmixia) was also expected to be associated with a *continuum* of morphological adaptations (e.g. shape, size, etc.) to specific environments. Nowadays, some morphological differences have been recorded between some Tanzanian regions. For example, it was observed that lions occupying woodlands displayed shorter manes compared to those living in plains [84]. Indeed, within the Selous GR (Cluster 1), a landscape dominated by the Miombo forest [85], lions seem to display a smaller mane as compared to the lions occupying savanna-type habitats even though this is still under investigation (P. Chardonnet, pers. comm.). Similar results were highlighted in the study of West & Packer [84], showing that adult males born in the Serengeti woodlands had shorter manes as compared to those born on the Serengeti plains. While the mane growth may be influenced by different factors other than genetic (e.g. climatic, environmental, social) [86,87], and therefore may potentially be of less concern, the translocation of animals between these distinct environments may be challenging, and even detrimental by leading to the loss of other specific local adaptations. The present findings therefore raise the question on how to best manage each lion population. Although the Selous GR was shown to display the largest pairwise *F*_*ST*_ indices with the other clusters, it also displayed the lowest *F*_*IS*_ value, while it is geographically connected through corridors to Niassa (Mozambique) and Mikumi NP (Tanzania). Moreover, a recent study demonstrated that maintaining a population size of at least 50 to 100 prides in a continuous ecosystem should avoid inbreeding depression within lions [88]. Based on these estimations and our present results, it seems that this cluster may be considered as presently sustainable.

Nevertheless, all three clusters have undergone recent demographic contraction, as supported by the census records. It would therefore be very important to avoid any further fragmentation within any of the identified clusters. Indeed, as underlined by Dolrenry *et al*. [89], lions have relatively weak dispersal capabilities, especially within an environment dominated by humans, with males generally mainly moving into neighboring territories close to their birth place [90]. Further fragmentation could lead to greater loss of connectivity between a mosaic of protected areas, and therefore of gene flow, which could in turn lead to rapid loss of genetic variability over time. Continuous future monitoring of these populations would be highly recommended to detect any risk of reduction of their fitness at an early stage [91].

The present results should also be taken into account when delimiting the LCUs: the 5 current LCUs defined for Tanzania in 2006 do not exactly correspond to the 3 identified clusters based on molecular markers. While a revision may be of interest, it is clear that for the conservation of the species, continuous monitoring on the largest possible sample would generate accurate information on the genetic health of Tanzanian lion populations and allow action to be rapidly taken whenever necessary [91].

## Conclusions

The present study supported assumptions that both ancient (over thousands of years) and recent (over the last century) population fragmentation has had an impact on the current genetic structure of the African lion, leading to the identification of two lineages at a continental scale (distinct management units or even subspecies), and of three genetic clusters in Tanzania. The results highlighted low levels of genetic differentiation between each Tanzanian cluster, as well as high genetic diversity and low inbreeding depression risks for each of them. Since human pressure between the three identified clusters is expected to increase in the near future, it is necessary to initiate appropriate management practices to ensure long-term conservation of African mammal diversity. In order to mitigate further genetic erosion, this should always be done while considering the environmental, behavioral, genetic and conservation related features of the concerned species.

## Supporting information

S1 Document Statistical analyses details and results of the *cytb* tree and network reconstruction, as well as the *cytb* genetic diversities estimations. (DOCX)

S1 Fig Phylogenetic tree reconstruction including all 17 haplotypes identified within the *P*. *leo* species. The tree was constructed with the maximum likelihood (ML) method using PhyML v3.0. Bootstrap support (above 800) are indicated on the branches. Orange: West-Central lineage, blue: East-Southern lineage.
(TIF)

S2 Fig Results of the Bayesian clustering analysis with Structure v2.3.4 software performed on the SNP database, reporting the Δ*K* values calculated according to Evanno *et al*. [55] with the CLUMPAK web server. (A) refers to the analysis conducted at the continental scale, including all samples (*K* = 2), while (B) reports the results for the analysis conducted at the Tanzanian country scale (*K* = 3).
(TIF)

S3 Fig FCA performed on the whole microsatellite dataset at the continental scale with Genetix v4.05. Yellow: Burkina Faso, CAR, Benin and Congo samples (West-Central lineage), blue: Tanzanian and South African samples (East-Southern lineage).
(TIF)

S4 Fig Correlogram of the average autocorrelation coefficient (*r*) for 18 distance classes of 50 km each. Dashed lines represent the 95% upper (U) and lower (L) bounds of the null distribution assuming no spatial structure. Error bars represent the 95% confidence intervals around *r*.
(TIF)

S5 Fig Geographic distribution of the three Cytochrome *b* haplotypes found in Tanzania (N = 38 samples). It is worth noting that at the Tanzanian country scale, some structures could also be highlighted based on the *cytb* haplotype distribution, as displayed on the present figure. Three distinct mitochondrial haplotypes (hap2, 13 and 17; S3 Table) were identified within a subset of 38 male samples, covering the same area as that of the individuals genotyped for SNPs. Nevertheless, the *cytb* haplotype organization was not similar to the observed structuring based on the SNP database, and instead depicted a more ancient evolutionary history. Hap2 and 13 were also recorded in Zambia, Kenya, Botswana and South Africa. Red: Hap2; green: Hap13; yellow: Hap17 (see reference in S3 Table).
(TIF)

S6 Fig Neighbor-joining tree reconstructed with Tassel v5.2.15 based on the SNPs database. Sample colors were attributed according to the STRUCTURE assignment posterior probabilities (Fig 5). BUR: Burkina Faso, CAR: Central African Republic, Green dot: root position.
(TIF)

S7 Fig Estimation of the number of populations based on microsatellite data, analysed by STRUCTURE. **(A)** Probability of successive partitions of the data into an increasing number of clusters obtained at the continental scale. **(B)** Probability of successive partitions of the data into an increasing number of clusters obtained at the Tanzanian scale. **(C)** Population structure of the lion populations from Tanzania into a partitioning for the modal solution K = 1 to K = 5. Each individual is represented by a thin vertical line divided into K coloured segments representing the probability of membership of this individual to the K clusters.
(TIF)

S1 Table List of the collected samples included in the present study. The table summarizes the sample origin (country and sampling locality), the number of samples collected at each locality, and gives a reference ID to Fig 1.
(DOCX)

S2 Table Details of the four microsatellite mixes designed within the present study. The primers were initially described by Menotti-Raymond *et al*. [39] for the *Felis catus* species. The two last columns report the expected allele sizes and heterozygosities.
(DOCX)

S3 Table List of *P*. *leo* haplotypes identified in the present study, including details about the geographic locations, corresponding lineage, number of samples included and new GenBank accession numbers. Numbers marked in bold represent the newly sequenced *cytb* gene. (?) indicates an uncertain sample origin, e.g. samples collected in zoos. CAR stands for Central African Republic, DRC for Democratic Republic of Congo.
(DOCX)
