# Supplementary material for: A genome-wide data assessment of the African lion (Panthera leo) population genetic structure and diversity in Tanzania
Source: PLoS One. 2018 Nov 7;13(11):e0205395. doi: 10.1371/journal.pone.0205395 (PMC6221261; doi:10.1371/journal.pone.0205395)
Supplement: S1 Document — (DOCX) [file pone.0205395.s001.docx]

*S7.1 Cytb tree and network reconstruction*

Our *cytb* database was supplemented with 74 *P. leo* *cytb* sequences from previous studies (GenBank accession numbers: GU131164-GU131185, AY781195-AY781210, DQ018993-DQ018996, DQ022291-DQ022301, AF384809-AF384818, KC495048- KC495058) [1–3]. Three *cytb* sequences of *Panthera tigris* were included as outgroup (KC495059, AF053051, AF053047). Haplotypes were determined using DNaSP v5.10.01 [4]. A maximum-likelihood (ML) haplotype tree was constructed using the HKY85 substitution model with PhyML v3.0 [5]. The nucleotide substitution model that best fit the data was identified with the web application FindModel ([www.hiv.lanl.gov](http://www.hiv.lanl.gov)) [6]. Bootstrap analysis (1,000 replicates) was used to estimate the robustness of the tree branches. A haplotype minimum spanning network was then reconstructed using the Minspnet algorithm implemented in Arlequin v3.5, using default settings [7]. At our study scale, networks were considered preferable to trees because they did not force haplotypes to occupy tip positions and allowed for multifurcations in the topology.

*S7.2 Cytb genetic diversities*

*Cytb* nucleotide (*π*) and haplotype (*h*) diversities and their standard deviations, as well as the net distance between lineages, were calculated using DNaSP v5.10.01 [4]. The demographic history was also inferred by testing the departure from neutrality using Fu’s *Fs* and Tajima’s *D* statistics in DNaSP. An exact test of population differentiation of pairwise weighted mean *F_ST_* [8] was performed using the same software (10,000 permutations for significance).

Cytochrome *b* haplotype diversity was shown to be greater in the East-Southern lineage (*h* = 0.809) as compared to the West-Central lineage (*h* = 0.588) (Table 1). The fixation index between both lineages was of 0.670 (*p*-value < 0.05), indicating that they were well differentiated. The net genetic divergence between the two groups was 0.7%.

**Table 1. Haplotype (*h*) and nucleotide (*π*) diversities computed for the two main lineages based on the *cytb* sequences.**

|  | | | **GENETIC DIVERSITY** | |
| --- | --- | --- | --- | --- |
| **LINEAGE** | N | N_h_ | *h* (SD) | *π* (SD) |
| **WEST-CENTRAL** | 49 | 4 | 0.588 (0.061) | 0.002 (0.001) |
| **EAST-SOUTHERN** | 79 | 13 | 0.809 (0.034) | 0.004 (0.002) |

N: sample size, N_h_: haplotype number.

References

1. Bertola LD, van Hooft WF, Vrieling K, Uit de Weerd DR, York DS, Bauer H, et al. Genetic diversity, evolutionary history and implications for conservation of the lion (Panthera leo) in West and Central Africa. J Biogeogr. 2011;38(7):1356–67.

2. Dubach J, Patterson BD, Briggs MB, Venzke K, Flamand J, Stander P, et al. Molecular genetic variation across the southern and eastern geographic ranges of the African lion, Panthera leo. Conserv Genet. 2005;6(1):15–24.

3. Dubach JM, Briggs MB, White PA, Ament BA, Patterson BD. Genetic perspectives on “Lion Conservation Units” in Eastern and Southern Africa. Conserv Genet. 2013;14(4):741–55.

4. Librado P, Rozas J. DnaSP v5: a software for comprehensive analysis of DNA polymorphism data. Bioinformatics. 2009;25(11):1451–2.

5. Guindon S, Gascuel O. A simple, fast, and accurate algorithm to estimate large phylogenies by maximum likelihood. Syst Biol. 2003;52(5):696–704.

6. Posada D, Crandall KA. Selecting the best-fit model of nucleotide substitution. Syst Biol. 2001;50(4):580–601.

7. Excoffier L, Lischer HEL. Arlequin suite ver 3.5: A new series of programs to perform population genetics analyses under Linux and Windows. Mol Ecol Resour. 2010;10(3):564–7.

8. Weir BS, Cockerham CC. Estimating F-Statistics for the analysis of population structure. Evolution (N Y). 1984;38(6):1358–70.
