# Supplementary figures and images for: A genome-wide data assessment of the African lion (Panthera leo) population genetic structure and diversity in Tanzania
Source: PLoS One. 2018 Nov 7;13(11):e0205395. doi: 10.1371/journal.pone.0205395 (PMC6221261; doi:10.1371/journal.pone.0205395)

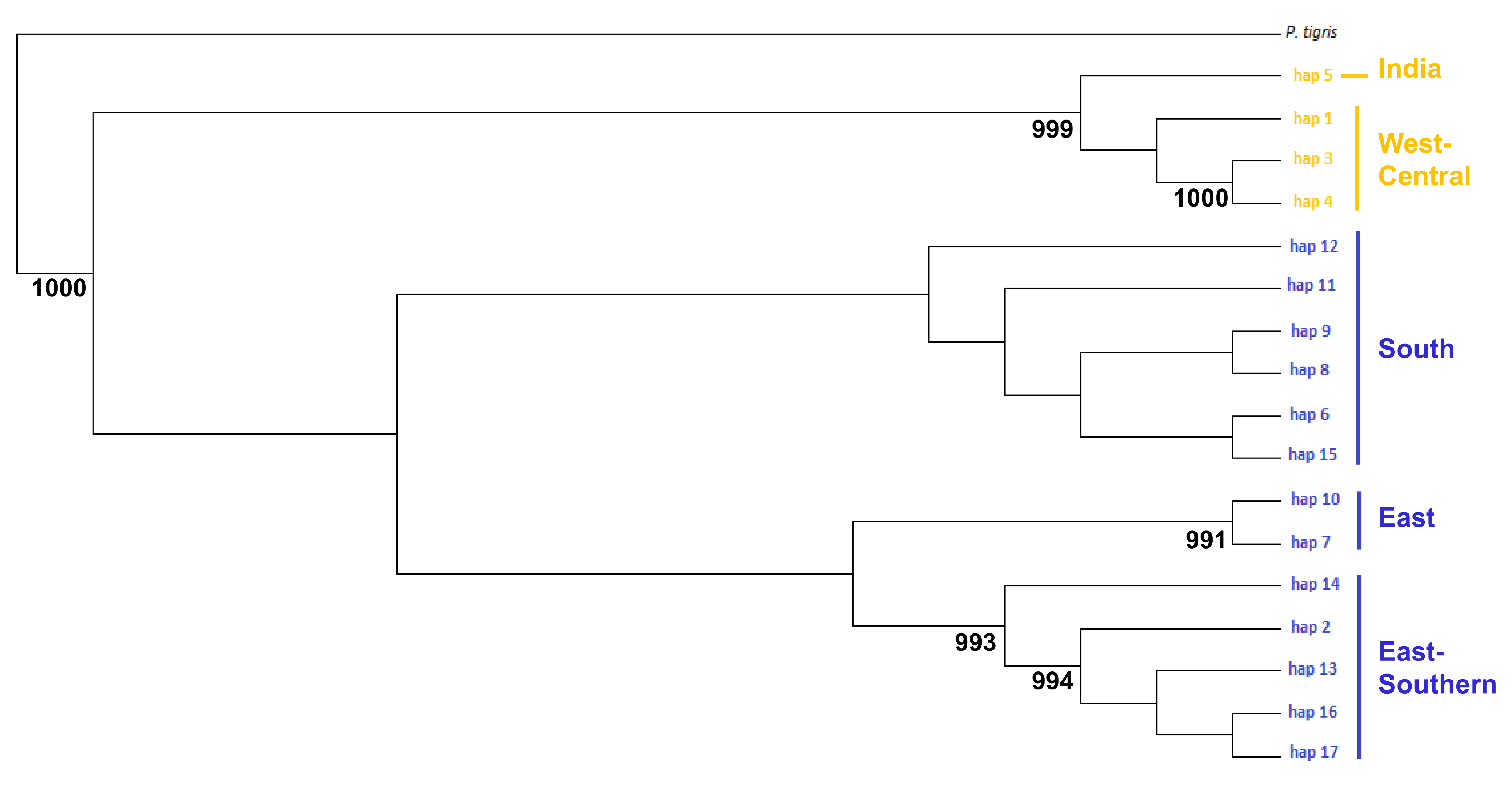

Supplement: S1 Fig — The tree was constructed with the maximum likelihood (ML) method using PhyML v3.0. Bootstrap support (above 800) are indicated on the branches. Orange: West-Central lineage, blue: East-Southern lineage. (TIF) [file pone.0205395.s002.tif]

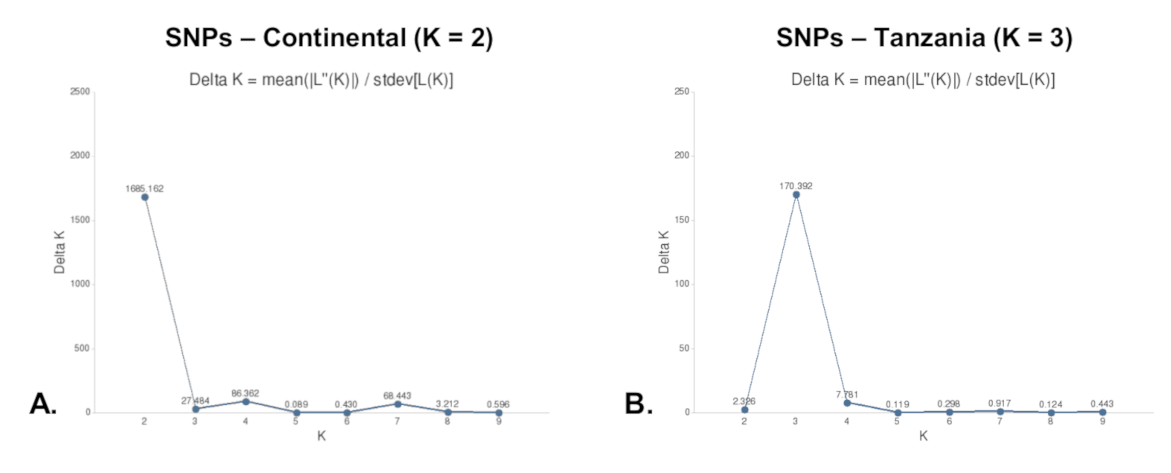

Supplement: S2 Fig — (A) refers to the analysis conducted at the continental scale, including all samples (K = 2), while (B) reports the results for the analysis conducted at the Tanzanian country scale (K = 3). (TIF) [file pone.0205395.s003.tif]

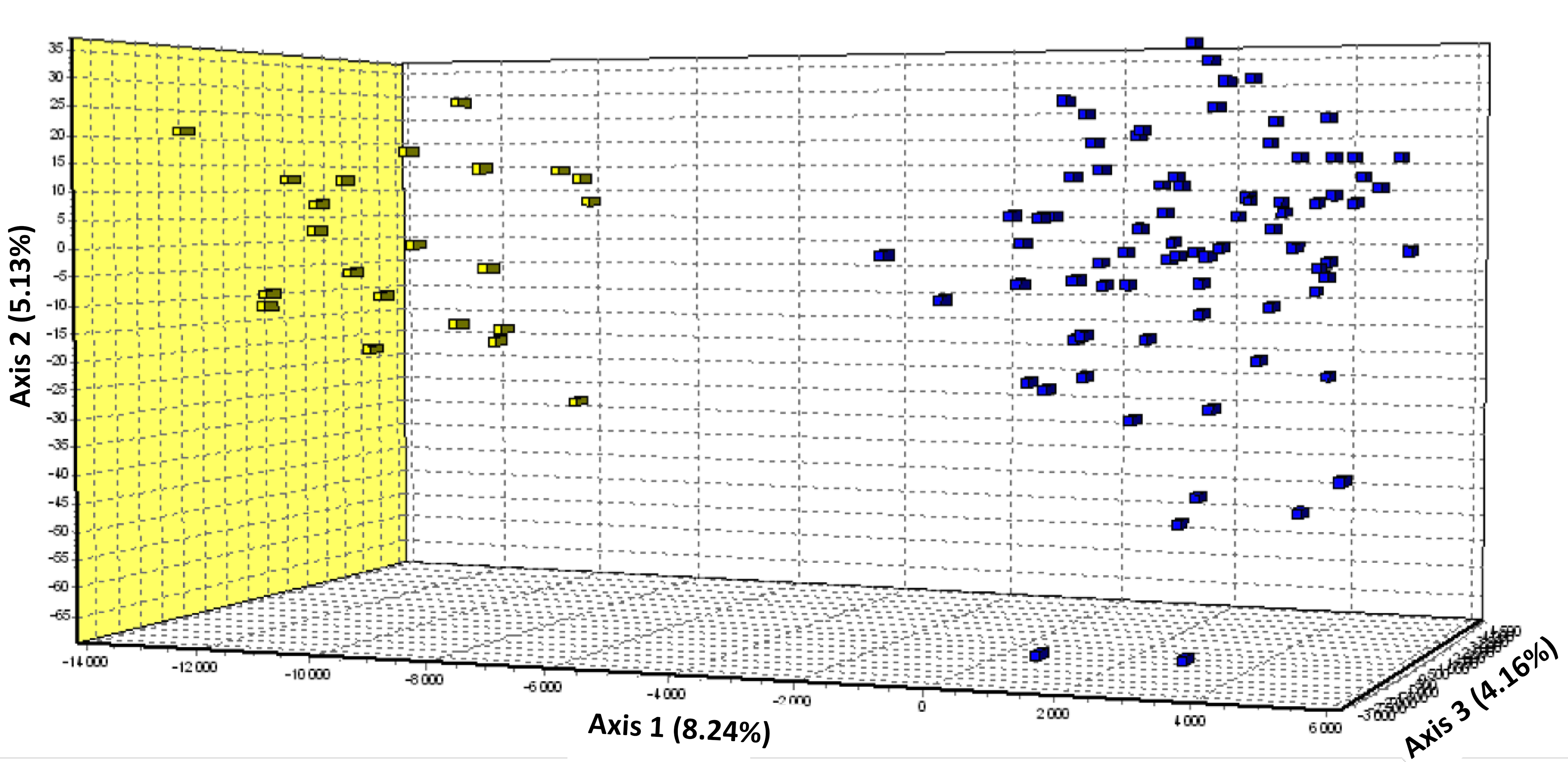

Supplement: S3 Fig — Yellow: Burkina Faso, CAR, Benin and Congo samples (West-Central lineage), blue: Tanzanian and South African samples (East-Southern lineage). (TIF) [file pone.0205395.s004.tif]

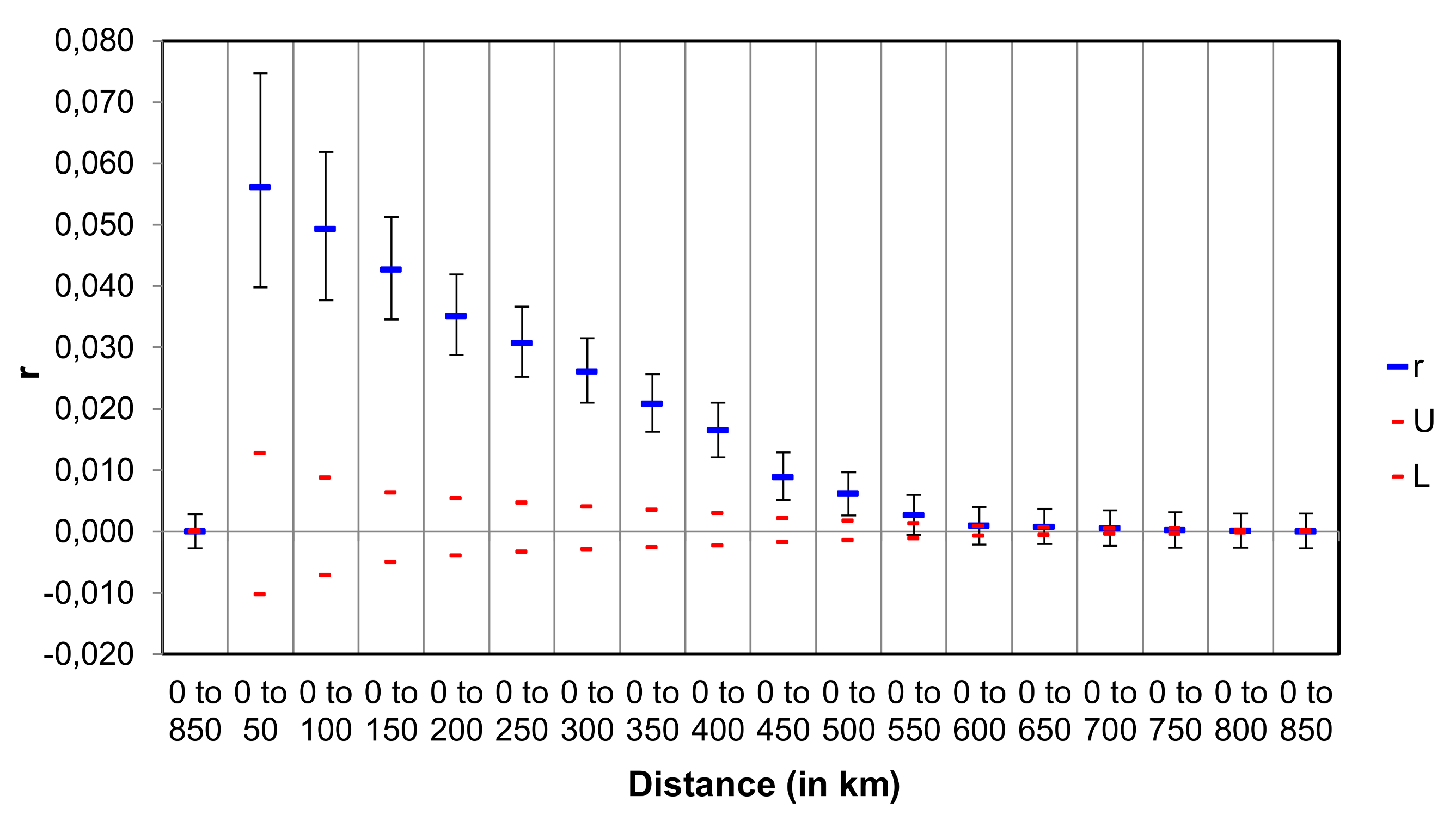

Supplement: S4 Fig — Dashed lines represent the 95% upper (U) and lower (L) bounds of the null distribution assuming no spatial structure. Error bars represent the 95% confidence intervals around r. (TIF) [file pone.0205395.s005.tif]

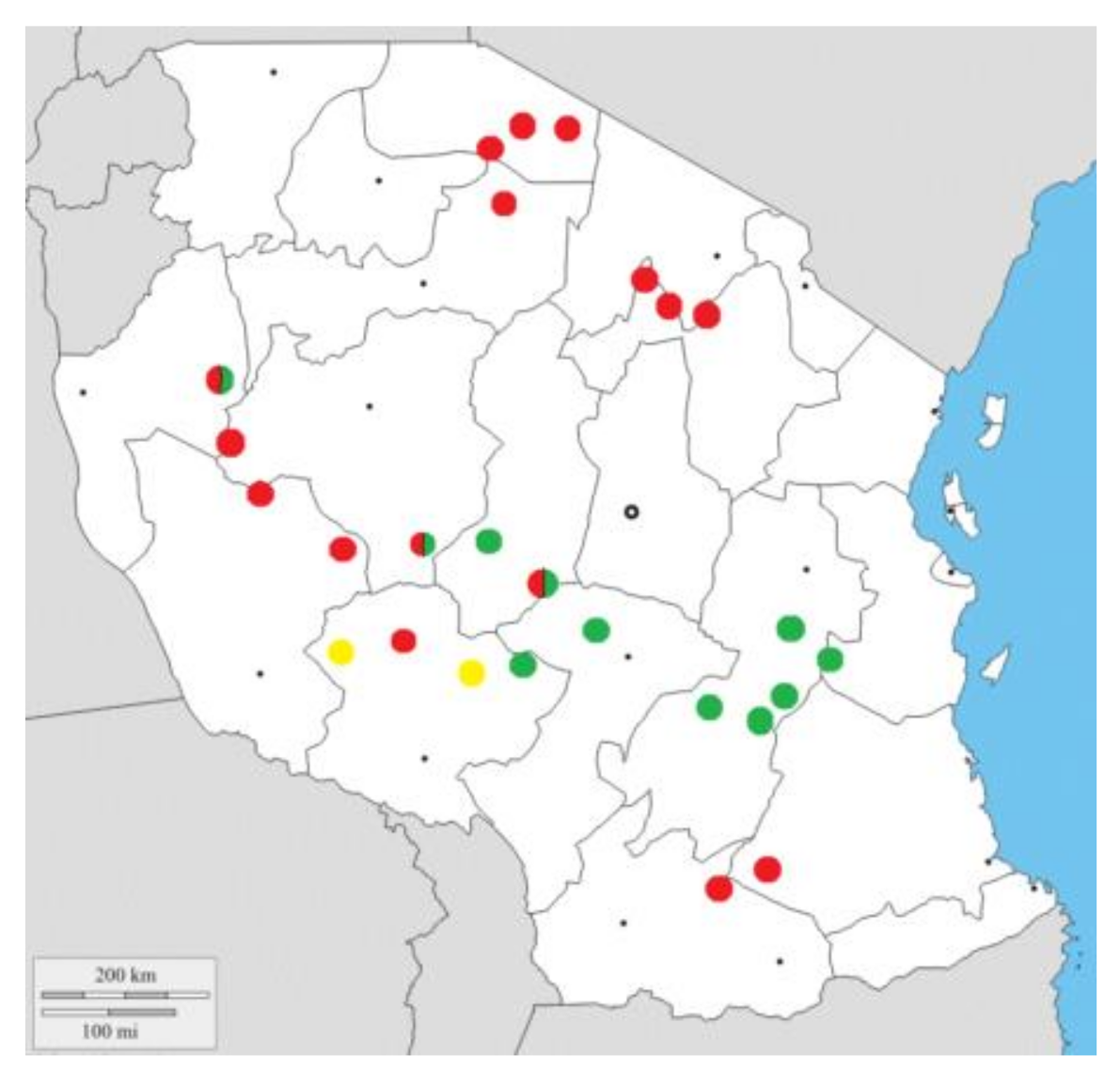

Supplement: S5 Fig — It is worth noting that at the Tanzanian country scale, some structures could also be highlighted based on the cytb haplotype distribution, as displayed on the present figure. Three distinct mitochondrial haplotypes (hap2, 13 and 17; S3 Table) were identified within a subset of 38 male samples, covering the same area as that of the individuals genotyped for SNPs. Nevertheless, the cytb haplotype organization was not similar to the observed structuring based on the SNP database, and instead depicted a more ancient evolutionary history. Hap2 and 13 were also recorded in Zambia, Kenya, Botswana and South Africa. Red: Hap2; green: Hap13; yellow: Hap17 (see reference in S3 Table). (TIF) [file pone.0205395.s006.tif]

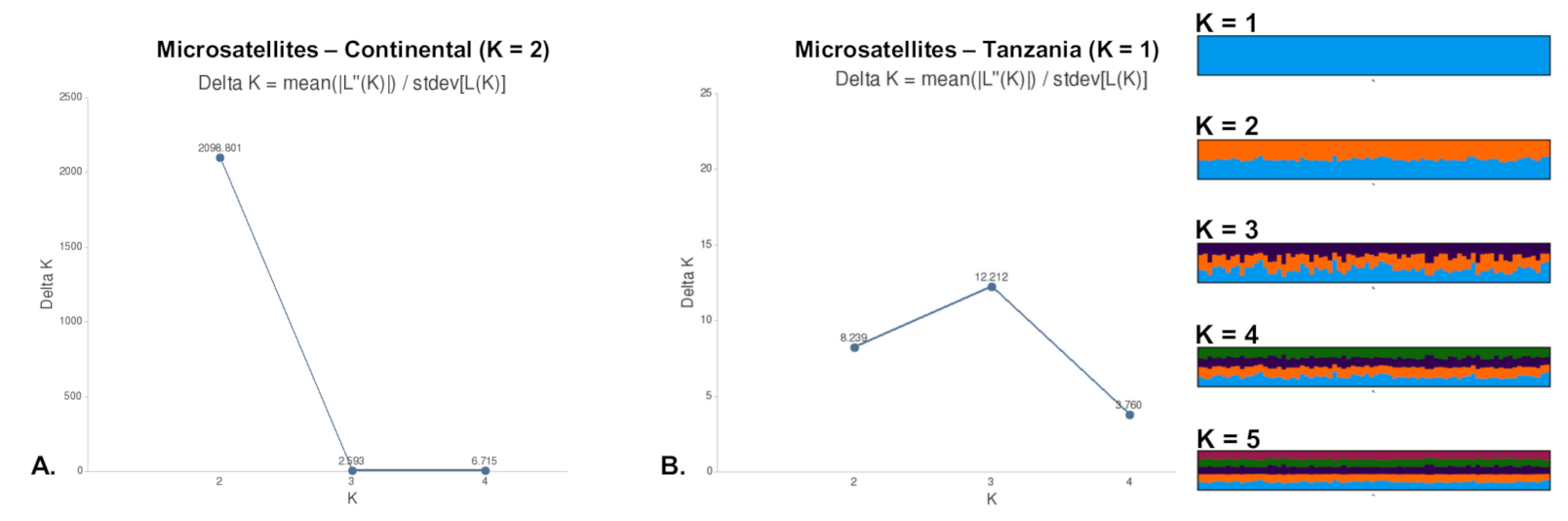

Supplement: S7 Fig — (A) Probability of successive partitions of the data into an increasing number of clusters obtained at the continental scale. (B) Probability of successive partitions of the data into an increasing number of clusters obtained at the Tanzanian scale. (C) Population structure of the lion populations from Tanzania into a partitioning for the modal solution K = 1 to K = 5. Each individual is represented by a thin vertical line divided into K coloured segments representing the probability of membership of this individual to the K clusters. (TIF) [file pone.0205395.s008.tif]
