# Supplementary material for: A genome-wide data assessment of the African lion (Panthera leo) population genetic structure and diversity in Tanzania
Source: PLoS One. 2018 Nov 7;13(11):e0205395. doi: 10.1371/journal.pone.0205395 (PMC6221261; doi:10.1371/journal.pone.0205395)
Supplement: S1 Table — The table summarizes the sample origin (country and sampling locality), the number of samples collected at each locality, and gives a reference ID to Fig 1. (DOCX) [file pone.0205395.s009.docx]

**S1 Table. List of the collected samples included in the present study.**

| **Country** | **Sampling locality** | **Fig 1. ID** | **N** |
| --- | --- | --- | --- |
| Tanzania | Burunge | A - 1 | 1 |
| Tanzania | Chunya Lukwati | A - 2 | 1 |
| Tanzania | Inyonga West | A - 3 | 1 |
| Tanzania | Rukwa lake | A - 4 | 2 |
| Tanzania | Loliondo | A - 5 | 2 |
| Tanzania | Lokisale | A - 6 | 1 |
| Tanzania | Selous LR2 | A - 7 | 1 |
| Tanzania | Selous LU5 | A - 8 | 2 |
| Tanzania | Luganzo | A - 9 | 1 |
| Tanzania | Lukwati North | A - 10 | 4 |
| Tanzania | Lukwati South | A - 11 | 3 |
| Tanzania | Lunda Mkwambi North | A - 12 | 1 |
| Tanzania | Selous MA1 | A - 13 | 1 |
| Tanzania | Simanjiro Kitangire | A - 14 | 2 |
| Tanzania | Maswa Kimali | A - 15 | 1 |
| Tanzania | Maswa North | A - 16 | 3 |
| Tanzania | Mbarang’andu | A - 17 | 1 |
| Tanzania | Moyowosi South | A - 18 | 2 |
| Tanzania | Msima East | A - 19 | 1 |
| Tanzania | Msima West | A - 20 | 1 |
| Tanzania | Muhesi West | A - 21 | 1 |
| Tanzania | Rungwa Ikili | A - 22 | 1 |
| Tanzania | Rungwa Mpera | A - 23 | 3 |
| Tanzania | Rungwa Mzombe | A - 24 | 5 |
| Tanzania | Rungwa South | A - 25 | 2 |
| Tanzania | Rungwa Rungwa East | A - 26 | 2 |
| Tanzania | Kizigo Central | A - 27 | 1 |
| Tanzania | Rungwa Rungwa West | A - 28 | 3 |
| Tanzania | Selous LR3 | A - 29 | 1 |
| Tanzania | Selous IH1 | A - 30 | 1 |
| Tanzania | Selous K1 | A - 31 | 1 |
| Tanzania | Selous LA1 | A - 32 | 1 |
| Tanzania | Selous LU1/LU2 | A - 33 | 1 |
| Tanzania | Selous K3/LU4 | A - 34 | 1 |
| Tanzania | Selous N1 | A - 35 | 1 |
| Tanzania | Selous LR1 | A - 36 | 1 |
| Tanzania | Serengeti | A - 37 | 2 |
| Tanzania | Tarangire | A - 38 | 6 |
| Tanzania | Ugunda | A - 39 | 2 |
| Tanzania | Wembere South | A - 40 | 1 |
| Tanzania | Selous unspecified | A - NA | 5 |
| South Africa | Breeding Farm (potential origin: Kruger National Park) | B | 3 |
| Central African Republic | Bamingui-Bangoran | C | 1 |
| Central African Republic | Goumbiri | C | 1 |
| Central African Republic | Koukourou-Bamingui | C | 1 |
| Central African Republic | Idongo da Bangoran | C | 1 |
| Congo | Odzala | D | 3 |
| Benin | Atakora-Mékrou | E | 1 |
| Burkina Faso | Koakrana | F | 2 |
| Burkina Faso | Konkombouri | F | 3 |
| Burkina Faso | Kourtiagou | F | 2 |
| Burkina Faso | Ougarou | F | 2 |
| Burkina Faso | Pagou-Tandougou | F | 5 |
| Burkina Faso | Pama | F | 5 |
| Burkina Faso | Singou | F | 1 |
| **TOTAL** |  |  | **105** |

The table summarizes the sample origin (country and sampling locality), the number of samples collected at each locality, and gives a reference ID to Fig 1.
