# Supplementary material for: A genome-wide data assessment of the African lion (Panthera leo) population genetic structure and diversity in Tanzania
Source: PLoS One. 2018 Nov 7;13(11):e0205395. doi: 10.1371/journal.pone.0205395 (PMC6221261; doi:10.1371/journal.pone.0205395)
Supplement: S2 Table — The primers were initially described by Menotti-Raymond et al. [39] for the Felis catus species. The two last columns report the expected allele sizes and heterozygosities. (DOCX) [file pone.0205395.s010.docx]

**S2 Table. Details of the four microsatellite mixes designed within the present study.**

| **MIX** | **MICROSATELLITE** | **PRIMERS** | **FLUOROCHROME** | **EXPECTED ALLELE SIZES** | **EXPECTED HETEROZYGOSITY** |
| --- | --- | --- | --- | --- | --- |
| **1** | **FCA014** | F : AGCCAAGAGGGGAAACAAACT  R : TGTCACAAATGGCAGGTTGT | VIC | 160 – 190 | 0.82 |
|  | **FCA030** | F : CTGTTGGAACTGGGAGTAAAGG  R : ACACATTTTCTCTGCCCCC | FAM | 120 – 142 | 0.82 |
|  | **FCA045** | F : TGAAGAAAAGAATCAGGCTGTG  R : GTATGAGCATCTCTGTGTTCGTG | NED | 146 – 160 | 0.85 |
|  | **FCA094** | F : TCAAGCCCCATTTTACCTTC  R : CACCTGAGCCAAAGGCTATC | PET | 215 – 237 | 0.90 |
| **2** | **FCA026** | F : GGAGCCCTTAGAGTCATGCA  R : TGTACACGCACCAAAAACAA | NED | 136 – 154 | 0.85 |
|  | **FCA191** | F : TCCTGTTCCTATTCACCCTACA  R : GCATGGCACTTTTGTTGAGA | VIC | 137 – 147 | 0.76 |
| **3** | **FCA096** | F : CACGCCAAACTCTATGCTGA  R : CAATGTGCCGTCCAAGAAC | FAM | 184 – 224 | 0.76 |
|  | **FCA132** | F : ATCAAGGCCAACTGTCCG  R : GATGCCTCATTAGAAAAATGGC | NED | 137 – 153 | 0.57 |
|  | **FCA187** | F : CCAACTGAACCACCCAGG  R : TGGATGGTTGTATTCTTCCTCA | PET | 162 – 172 | 0.80 |
| **4** | **FCA077** | F : GGCACCTATAACTACCAGTGTGA  R : ATCTCTGGGGAAATAAATTTTGG | FAM | 143 – 155 | 0.54 |
|  | **FCA126** | F : GCCCCTGATACCCTGAATG  R : CTATCCTTGCTGGCTGAAGG | PET | 139 – 145 | 0.71 |

The primers were initially described by Menotti-Raymond *et al.* [39] for the *Felis catus* species. The two last columns report the expected allele sizes and heterozygosities.
