# Supplementary material for: A genome-wide data assessment of the African lion (Panthera leo) population genetic structure and diversity in Tanzania
Source: PLoS One. 2018 Nov 7;13(11):e0205395. doi: 10.1371/journal.pone.0205395 (PMC6221261; doi:10.1371/journal.pone.0205395)
Supplement: S3 Table — Numbers marked in bold represent the newly sequenced cytb gene. (?) indicates an uncertain sample origin, e.g. samples collected in zoos. CAR stands for Central African Republic, DRC for Democratic Republic of Congo. (DOCX) [file pone.0205395.s011.docx]

**S3 Table. List of *P. leo* haplotypes identified in the present study, including details about the geographic locations, corresponding lineage, number of samples included and new GenBank accession numbers.**

| **HAPLOTYPES** | **COUNTRY** | **TOTAL NUMBER OF INDIVIDUALS** | **LINEAGE** | **GENBANK ACCESSION NUMBER** |
| --- | --- | --- | --- | --- |
| **Hap1** | Benin | **1** + 2 | West-Central | MG677918 |
|  | Burkina Faso | **11** | West-Central |  |
| **Hap2** | Kenya | 2 | East-Southern | / |
|  | Tanzania | **22** | East-Southern | MG677919 |
|  | Botswana | 3 | East-Southern | / |
|  | South Africa | 3 | East-Southern | / |
| **Hap3** | Cameroun | 1 | West-Central | / |
|  | Chad | 3 | West-Central | / |
| **Hap4** | Cameroun | 11 | West-Central | / |
|  | Chad | 1 | West-Central | / |
|  | Sudan | 1 | West-Central | / |
|  | CAR | **4** | West-Central | MG677920 |
|  | DRC (?) | 1 | West-Central | / |
|  | Angola (?) | 1 | East-Southern | / |
|  | Morocco (?) | 10 | West-Central | / |
|  | Senegal | 2 | West-Central | / |
| **Hap5** | India | 4 | North | / |
| **Hap6** | Namibia | 2 | East-Southern | / |
| **Hap7** | Somalia (?) | 3 | East-Southern | / |
|  | Kenya | 2 | East-Southern | / |
|  | Uganda | 1 | East-Southern | / |
| **Hap8** | South Africa | 4 | East-Southern | / |
| **Hap9** | South Africa | 1 | East-Southern | / |
| **Hap10** | Ethiopia | 4 | East-Southern | / |
| **Hap11** | Namibia | 2 | East-Southern | / |
| **Hap12** | Namibia | 4 | East-Southern | / |
|  | Botswana | 2 | East-Southern | / |
| **Hap13** | Tanzania | **14** | East-Southern | MG677921 |
|  | Zambia | 1 | East-Southern | / |
| **Hap14** | Zambia | 1 | East-Southern | / |
| **Hap15** | Namibia | 1 | East-Southern | / |
| **Hap16** | Namibia | 1 | East-Southern | / |
| **Hap17** | Tanzania | **2** | East-Southern | MG677922 |
| **TOTAL** |  | **54** + 74 = 128 |  |  |

Numbers marked in bold represent the newly sequenced *cytb* gene. (?) indicates an uncertain sample origin, e.g. samples collected in zoos. CAR stands for Central African Republic, DRC for Democratic Republic of Congo.
